# Phylogenetic and Transcriptomic Analysis of Chemosensory Receptors in a Pair of Divergent Ant Species Reveals Sex-Specific Signatures of Odor Coding

**DOI:** 10.1371/journal.pgen.1002930

**Published:** 2012-08-30

**Authors:** Xiaofan Zhou, Jesse D. Slone, Antonis Rokas, Shelley L. Berger, Jürgen Liebig, Anandasankar Ray, Danny Reinberg, Laurence J. Zwiebel

**Affiliations:** 1 Department of Biological Sciences, Vanderbilt University, Nashville, Tennessee, United States of America; 2 Departments of Cell and Developmental Biology, Genetics, and Biology, University of Pennsylvania, Philadelphia, Pennsylvania, United States of America; 3 School of Life Sciences, Arizona State University, Tempe, Arizona, United States of America; 4 Department of Entomology, University of California Riverside, Riverside, California, United States of America; 5 Howard Hughes Medical Institute, Department of Biochemistry, New York University School of Medicine, New York, New York, United States of America; Yale University, United States of America

## Abstract

Ants are a highly successful family of insects that thrive in a variety of habitats across the world. Perhaps their best-known features are complex social organization and strict division of labor, separating reproduction from the day-to-day maintenance and care of the colony, as well as strict discrimination against foreign individuals. Since these social characteristics in ants are thought to be mediated by semiochemicals, a thorough analysis of these signals, and the receptors that detect them, is critical in revealing mechanisms that lead to stereotypic behaviors. To address these questions, we have defined and characterized the major chemoreceptor families in a pair of behaviorally and evolutionarily distinct ant species, *Camponotus floridanus* and *Harpegnathos saltator*. Through comprehensive re-annotation, we show that these ant species harbor some of the largest yet known repertoires of odorant receptors (*Ors*) among insects, as well as a more modest number of gustatory receptors (*Grs*) and variant ionotropic glutamate receptors (*Irs*). Our phylogenetic analyses further demonstrate remarkably rapid gains and losses of ant *Ors*, while *Grs* and *Irs* have also experienced birth-and-death evolution to different degrees. In addition, comparisons of antennal transcriptomes between sexes identify many chemoreceptors that are differentially expressed between males and females and between species. We have also revealed an agonist for a worker-enriched OR from *C. floridanus*, representing the first case of a heterologously characterized ant tuning Or. Collectively, our analysis reveals a large number of ant chemoreceptors exhibiting patterns of differential expression and evolution consistent with sex/species-specific functions. These differentially expressed genes are likely associated with sex-based differences, as well as the radically different social lifestyles observed between *C. floridanus* and *H. saltator*, and thus are targets for further functional characterization. Our findings represent an important advance toward understanding the molecular basis of social interactions and the differential chemical ecologies among ant species.

## Author Summary

Chemical communication is an important factor in the regulation of social interaction in animals. The family of eusocial insects commonly known as ants offers an almost unique opportunity for examining the genetic basis for the chemosensory pathways that underlie ant sociality. In order to address this issue, we have manually and comprehensively reannotated the chemoreceptor repertoire in a pair of evolutionarily and behaviorally divergent ant species, *Camponotus floridanus* and *Harpegnathos saltator*. In addition, we have used next-generation RNA sequencing to examine the chemosensory receptor transcriptome between males and females within these species. Our analysis demonstrates rapid gene birth-and-death for the ant odorant and gustatory receptor gene families, as well as clear differences in the expression of particular subsets of chemoreceptor genes between males and females. Finally, we have begun to examine the odor space within these discrete social units by heterologous characterization of the first *C. floridanus* odorant receptor that also exhibits sex-specific differential expression. Taken together, our results provide a foundation for future studies of the genetic basis for the chemical signaling and chemical ecology underlying the dramatically different social lifestyles exhibited by these and other species of ants.

## Introduction

The family of insects commonly known as ants (family Formicidae) originated during the Cretaceous period, approximately 140 million years ago [1]. Since that time, they have established a global presence, with only the most remote locations lacking ant species [2]. Indeed, in some cases, such as lowland tropical rainforest canopies, ants have come to dominate the biomass [3], [4]. Their ecological success is reflected in the number and diversity of ants, of which there were 283 known genera [5].

There is a wide diversity in the behavior and morphology of different ant subfamilies that includes both the level and complexity of social organizations. For instance, *Camponotus floridanus* (the Florida Carpenter Ant), is a Formicine ant from the South-Eastern United States which belongs to one of the most globally prevalent ant genera [6]. These ants feature a rigid caste structure, with strict division of labor between the reproductive queens and the non-reproductive workers that is primarily regulated through pheromones [7], [8], [9]. Workers have a high threshold to lay eggs, and regulation of their reproduction through aggressive interactions does not occur [10]. Furthermore, the worker caste is divided into two classes: minor workers and major workers, which differ in size and morphology [2], [6]. On the other hand, *Harpegnathos saltator*, a predatory species of Ponerine ant endemic to India and Sri Lanka is characterized by a more flexible reproductive system. *H. saltator* colonies are relatively small (averaging 65 to 225 individuals, depending on season and region) [11], and queen to worker dimorphism is weak [11], [12]. When a *H. saltator* colony loses its queen, one or more of the workers will begin laying eggs and become functional reproductives (referred to as gamergates) [12] and this behavioral transition is initiated with strong aggressive interactions [13].

Sociality in ants is considered to be a simple model for complex behaviors in humans and other mammals [14]. The success of ants is thought to have arisen in large part from their well-developed eusociality, wherein individuals live together in colonies with one or several highly fertile female “queens” surrounded by a host of non-reproductive female “workers.” These workers then support and defend the queen and her progeny. The fact that the workers are the queen's own daughters is thought to provide the evolutionary advantage for the workers to protect and support the queen [6].

While it is generally accepted that a variety of chemical signals mediate many of the interactions between these castes, as well as interactions between individuals from competing colonies, there is great interest in determining the particular pheromones and their cognate molecular receptors that mediate these interactions [2]. It is likely that these semiochemicals are initially detected in peripheral sensory neurons by members of three major insect chemosensory receptor gene families: odorant receptors (*Ors*) [15], [16], [17], [18], [19], gustatory receptors (*Grs*) [15], [20], [21], [22], [23], and the more recently discovered variant ionotropic glutamate receptors (*Irs*) [24], [25], [26].

*Ors* and *Grs* belong to the same superfamily and both encode seven-transmembrane-domain proteins [17], [22]. *Ors* are mainly expressed in olfactory receptor neurons (ORNs) within sensory appendages such as antennae and maxillary palps, where they are responsible for the perception of volatile chemical signals [17], [19]. Conventional insect Ors (so-called “tuning” Ors) are associated with odorant specificity. They are typically highly divergent and their orthologous relationships are usually difficult to determine even within order (e.g. *Drosophila* vs. *Anopheles* [27], and *Nasonia* vs. *Apis* [28]). In contrast, one member of this gene family, which is now uniformly known as *Orco*, is both highly conserved across insect orders and widely expressed in a majority of ORNs [29], [30]. Orco is necessary and sufficient for the proper localization and retention of other tuning Ors at the dendritic membrane, and is required for proper function of tuning Ors [29], [31]. Rather than playing a role in odorant specificity, Orco forms an essential part of a heteromeric ion channel in cooperation with a tuning Or that is gated by its cognate odor ligand [32], [33], [34], [35], [36].

In contrast with the *Ors*, *Grs* are highly expressed in gustatory organs [20], [21], [22], and a large portion of these receptors respond to soluble tastants [37], [38], [39] and pheromones [40], [41], [42], leading to the “gustatory” designation for this group of chemoreceptors. However, there are some exceptions; for example, one unusual group of Grs respond to the volatile chemical carbon dioxide [43], [44], demonstrating that members of this receptor family are not necessarily limited to gustatory or pheromonal responses. This is further supported by the expression of some *Grs* in non-gustatory organs such as the arista and Johnston's organ [45].

*Irs* are homologous to ionotropic glutamate receptors (*iGluRs*) and thus are evolutionarily unrelated to *Ors* and *Grs* [24], [26]. The role of IRs as chemosensory receptors has recently been uncovered based on multiple lines of evidence, including their divergence from conventional iGluRs at sequence level and the expression of several Irs in chemosensory neurons [24]. While Irs are generally thought to mediate responses to acids and amines [25], members of this family of chemosensory receptors may also sense other classes of chemicals.

We hypothesize that the striking contrast between *C. floridanus*, with its strict queen-worker dimorphism and largely pheromone-regulated reproduction, and *H. saltator*, with its flexible reproductive system that is associated with behavioral and pheromonal regulation of reproduction, is correlated with distinctive semiochemical and chemoreceptor profiles, which in turn generate differences in their chemical ecologies. The same is likely to be true of caste- or sex-based differences in behavior within each species.

To test these hypotheses, we first developed a custom gene annotation pipeline to comprehensively describe the chemosensory receptor repertoires of *C. floridanus* and *H. saltator*. We then investigated the evolutionary patterns (e.g. gene gain-and-loss) of these chemosensory receptor genes, in order to gain insight on their functional diversification. Furthermore, we performed RNAseq analyses of caste- and sex-specific antennal transcriptomes to identity chemoreceptors that are differentially expressed between males/females and between species. We found multiple clades of chemosensory receptor genes that show differential expansion/contraction among ant species. In addition, a large number of chemosensory receptor genes exhibited sex-specific expression or male/female-enrichment. These chemosensory receptor genes exhibiting interesting evolutionary and expression patterns may have potentially contributed to the different chemical ecology between sexes/species. We also successfully identified agonists for two *Or* genes to further validate these annotations. The findings of this study inform us as to the genetic basis for the differences in chemical ecology between *C. floridanus* and *H. saltator*, as well as the potential role of chemosensory receptors in the biology and evolution of eusociality in ants.

## Results

### Annotation of *C. floridanus* and *H. saltator* chemosensory receptor genes

**Figure 1:**
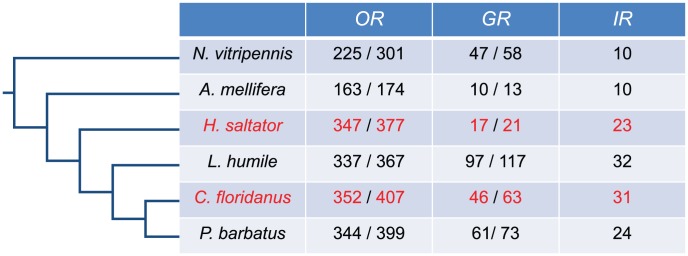
Annotation of *C. floridanus* and *H. saltator* chemosensory receptor genes. Number of *Or*, *Gr*, and *Ir* gene predictions in six hymenopteran species. For *Or* and *Gr* genes, the number to the right is the number of all gene models (coding for proteins longer than 300 aa in *C. floridanus* and *H. saltator*, or 200 aa in other species), while the number to the left is the number of seemingly intact gene models.

The automated genome annotations of *C. floridanus* and *H. saltator* revealed about 100 *Or* and about 10 *Gr* genes [46], which is substantially fewer than the number of *Or* and *Gr* genes in two other sequenced ant genomes (e.g. argentine ant: *Linepithema humile* [47], and harvester ant: *Pogonomyrmex barbatus* [48]; Figure 1). These low numbers were not surprising because the annotation of *Or*/*Gr* genes in other insect genomes has been difficult and usually requires extensive manual efforts [47], [48]. In order to address this potential discrepancy and comprehensively elucidate the genomic repertoire of chemosensory receptor genes in *C. floridanus* and *H. saltator*, we rigorously re-annotated *Or*, *Gr*, and *Ir* genes in these two ant species using a custom automated pipeline followed by careful manual inspection.

To maximize the sensitivity of our re-annotation, we collected reported *Or*, *Gr*, and *Ir* gene sequences from other sequenced Hymenoptera and insect relatives of *C. floridanus* and *H. saltator*, including *Apis mellifera*, *Acyrthosiphon pisum*, *Drosophila melanogaster*, *Nasonia vitripennis*, *L. humile,* and *P. barbatus.* These insect chemosensory receptor genes were used to identify putative *Or*/*Gr*/*Ir* coding regions within the *C. floridanus* and *H. saltator* genomes and to guide homology-based gene prediction. As a result, we discovered a large number of previously unannotated chemosensory receptor genes and corrected several previously reported gene models [46]. All these annotations were manually inspected in multiple sequences alignments to identify and correct for potential errors (e.g. missing exons, unrelated sequences). This analysis indicates that *C. floridanus* contains 407 putative *Or* coding loci, of which 352 loci encode intact *Or* genes, which is similar to those newly annotated in *H. saltator*, with 377 loci in total and 347 intact loci (all chemosensory receptor genes annotated in this study are available in Dataset S1). The number of *Ir* predictions is also similar between the two ants, with 31 *Ir* genes in *C. floridanus* and 23 in *H. saltator*. On the other hand, *C. floridanus* contains 46 intact *Gr* genes, which is significantly higher than the 17 intact *Gr* genes found in *H. saltator* (Figure 1). Moreover, all three families of chemosensory receptor genes exhibited high degrees of sequence divergence among family members (Table S1).

In addition to the chemosensory receptor genes listed above, we also found a large number of incomplete gene models in these two ant genomes. For example, in *C. floridanus* and *H. saltator*, there are respectively ∼100 and ∼80 *Or* gene models encoding proteins shorter than 300 amino acids. In parallel to the difference in intact *Gr* genes, only three fragmented *Gr* gene models were found in *H. saltator*, while *C. floridanus* has ∼30 short *Gr* genes. Close examination of their genomic sequences revealed two principal mechanisms apparently leading to these fragmented *Or*/*Gr* gene models: 1) the presence of multiple frame-shift mutations and premature stop-codons, suggesting that they represent pseudogenes; and 2) their locations around undetermined genomic regions (e.g. edges of contigs/scaffolds), indicative of incomplete assembly as expected from a draft genome. The latter mechanism explains about 80% of the incomplete gene models.

Furthermore, similar to other insects [28], [47], [48], [49], [50], [51], most chemosensory receptor genes are tandemly arrayed in the *C. floridanus* and *H. saltator* genomes. In both cases, about 75% of *Or* genes are located in gene clusters of 4 to about 40 genes, and these occur in 24 and 20 *Or* gene clusters (n≥4) in *C. floridanus* and *H. saltator*, respectively (Figure S1). Although to a lesser degree than the *Ors*, half of the *Gr* and *Ir* genes in both ants have at least one neighboring homolog.

### Phylogenetic analysis

To better understand the evolutionary history of chemosensory receptor genes in the two ant species, we performed Hymenoptera-wide phylogenetic analysis on each of the *OR*, *GR*, and *IR* gene families. Additional analyses including *D. melanogaster* and *Tribolium castaneum* showed that most relationships among hymenopteran and non-hymenopteran sequences were not resolved within the *OR* and *GR* families (see below). In this study, while they are generally categorized as belonging to the same receptor superfamily [22], we elected to analyze the *OR* and *GR* families separately due to their high level of divergence.

#### *OR* family

**Figure 2:**
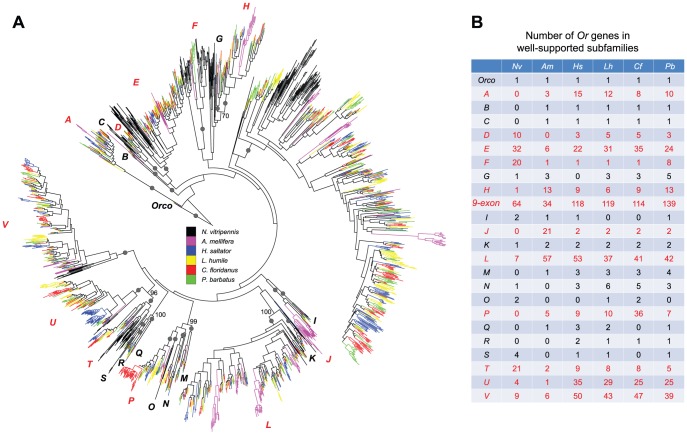
Phylogenetic relationships of Hymenoptera *Or* genes. (A) A maximum-likelihood tree of hymenopteran *Or* genes estimated by using RAxML with Le-Gascuel (LG) model. Reliability of internal nodes was evaluated by 100 bootstrap replicates. Grey round dots indicate well-supported subfamilies (bootstrap value ≥80). Bootstrap values ≥70 are shown for relationships among subfamilies. *Ors* in different species are color-coded as following: *N. vitripennis*, black; *A. mellifera*, purple; *H. saltator*, blue; *L. humile*, yellow; *P. barbatus*, green; and *C. floridanus*, red. Subfamilies with rapid changes in gene copy numbers are highlighted in red. (B) Numbers of hymenopteran *Or* genes in well supported subfamilies.

Our phylogenetic analysis of hymenopteran *Or* genes revealed a highly dynamic evolutionary history of this gene family featuring rapid gene birth and death (Figure 2A). Due to the rapid divergence of *Or* genes (average amino acid distance = 2.56; overall protein sequence identity = 19.45%), most deep relationships in the *OR* phylogeny lacked support (see Figure S2 and Dataset S2 for the full version of *OR* phylogeny with gene names and bootstrap values). In spite of this, we found 24 well-supported clades (referred to as subfamilies; *A*-*V*, *Orco*, and *9-exon* in Figure 2), each potentially representing one *Or* gene copy in the common ancestor of Hymenoptera (also see Figure S3 for the *OR* phylogeny with *D. melanogaster* and *T. castaneum* sequences). These subfamilies exhibited vastly different patterns of expansion/contraction, which can be divided into three types (Figure 2A, 2B): 1) strict single-copy representation in each of the six analyzed hymenopterans was observed for the *Orco* subfamily, which is the only *Or* gene with clear orthologous relationships throughout insects [15], [28], [50], [51], [52], [53], [54] (Figure S3); 2) 11 subfamilies showed either gene loss only, or a limited number of gene duplication events (e.g. *B* and *C*); 3) the remaining 12 subfamilies had experienced substantial expansions within and/or shared by hymenopteran lineages (e.g. *A* and *D*).

In particular, the most dramatic expansion was found in the subfamily composed of *Or* genes with 9 exons (Figure 2A, 2B). This *9-exon* subfamily encompasses more than 30% of the entire repertoires of *Or* genes in the six hymenopterans, which is in agreement with previous observations in other ants [47], [48]. Furthermore, our analysis revealed highly dynamic *Or* evolution within these subfamilies; subclades were often differentially expanded and/or contracted in different species and rapid expansions were usually accompanied by frequent gene losses (Figure S4).

As described above, most *Or* genes in *C. floridanus* and *H. saltator* were found in tandem arrays in their respective genomes. Our phylogenetic results provided further evidence that these clustered *Or* genes were derived from tandem whole-gene duplication events. Moreover, more than 60% of all tandem duplicates in the two ants were due to lineage-specific expansions, while the others were generated during or even before the divergence of Hymenoptera. For example, the neighboring *Or* genes on *C. floridanus* scaffold538 and *H. saltator* scaffold105 belonged to four different subfamilies, suggesting that these two gene clusters were established before the divergence of the six hymenopterans, and underwent further expansion within each lineage (Figure S5).

Although Hymenoptera-wide orthology of *Or* genes may have been obscured by rapid gene gain and loss, we were still able to identify several clear 1-to-1 relationships among Formicidae. In total, we found 35 ant-specific clades that are composed of a single copy of *Or* gene from each of the four ants. Among these, genes in 30 clades are located in gene clusters while all others occur as singletons. A chi-square test showed that neither tandem duplicates nor singletons are significantly enriched in the 35 orthologous gene clades (*p*-value = 0.05).

#### *GR* family

**Figure 3:**
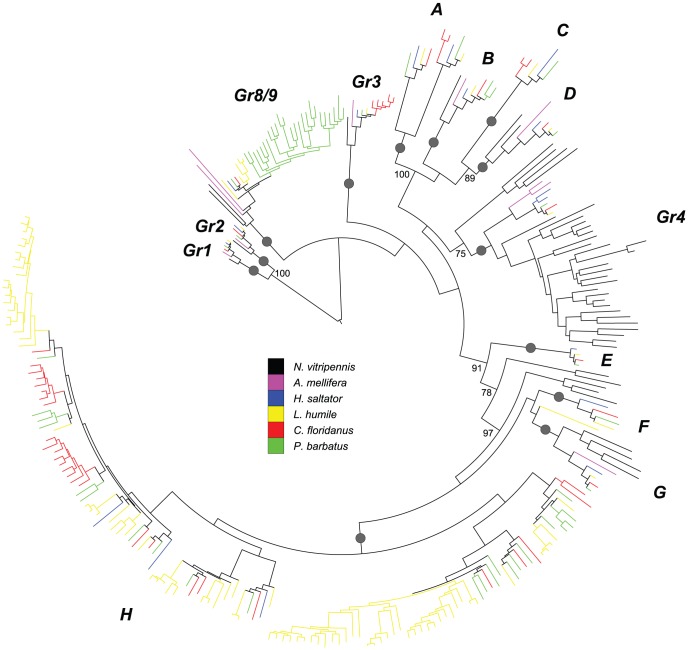
Phylogenetic relationships of Hymenoptera *Gr* genes. A maximum-likelihood tree of hymenopteran *Gr* genes estimated by using RAxML with Le-Gascuel (LG) model. Reliability of internal nodes was evaluated by 100 bootstrap replicates. Grey round dots indicate well-supported subfamilies (bootstrap value ≥80). Bootstrap values ≥70 are shown for relationships among subfamilies. Subfamilies showing interesting evolutionary patterns are named after the orthologs in *N. vitripennis* and *A. mellifera*. The other subfamilies are named as *A*–*H*. *Grs* in different species are color-coded as following: *N. vitripennis*, black; *A. mellifera*, purple; *H. saltator*, blue; *L. humile*, yellow; *P. barbatus*, green; and *C. floridanus*, red.

Similar to the *OR* family, the *GR* phylogeny provided evidence for birth-and-death evolution in this family (Figure 3; see Figure S6 and Dataset S3 for the full version of *GR* phylogeny with gene names and bootstrap values). Within the *Gr* phylogeny, 13 well-supported subfamilies were found, most of which were likely generated by ancestral duplications before the divergence of Hymenoptera (although the precise relationships among them remained unresolved). Within Hymenoptera, ancestral duplications and/or lineage-specific expansions were found in most subfamilies, except for the *GR1* and *GR2* subfamilies. Indeed, significant lineage-specific expansions of ant *Gr* genes include the *GR3* (*L. humile* and *P. barbatus*) and *GR8/9* (*C. floridanus*) subfamilies. The most dramatic expansion was observed in an ant-specific subfamily which underwent multiple rounds of amplifications before and after the separation of *H. saltator*, especially within *C. floridanus* and *L. humile*. In contrast to the other three ants, *H. saltator* specific duplication was only observed once (in the *GR4* subfamily), which explains the low number of *Gr* genes in this species. Moreover, our *GR* phylogeny showed that the formation of *Gr* gene clusters was likely due to tandem duplication, highlighting the importance of this duplication mechanism in the evolution of chemosensory receptor genes.

Among the *Grs*, orthologs of the known sugar receptor genes (*Gr1* and *Gr2*) [55], [56], [57], [58] and another insect-wide conserved *Gr*, *D. melanogaster Gr43a* (*Gr3*) [28], [53], [54], were observed in all of the species examined (also see Figure S7 for the *GR* phylogeny with *D. melanogaster* and *T. castaneum* sequences). However, no orthologs of the well-described dipteran carbon dioxide (CO_2_) receptor genes [43], [59] were found (Figure S7), consistent with the proposed loss of dipteran CO_2_ receptors in the ancestor of Hymenoptera [60]. Interestingly, it is known that the ability to perceive CO_2_ is present in ants [61], suggesting that different receptor genes are involved.

#### *IR* family

**Figure 4:**
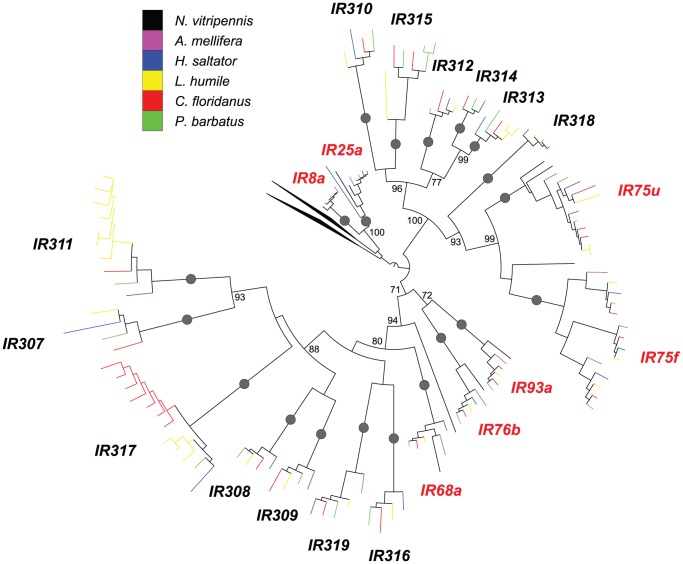
Phylogenetic relationships of Hymenoptera *Ir* genes. A maximum-likelihood tree of hymenopteran *Ir* genes estimated by using RAxML with Le-Gascuel (LG) model. Reliability of internal nodes was evaluated by 100 bootstrap replicates. Grey round dots indicate well-supported clades of “antennal” or “divergent” *IRs* (bootstrap value ≥80). Bootstrap values ≥70 are shown for relationships among the well supported clades. Subfamilies are named after the orthologs in *L. humile* and *P. barbatus*. *Irs* in different species are color-coded as following: *N. vitripennis*, black; *A. mellifera*, purple; *H. saltator*, blue; *L. humile*, yellow; *P. barbatus*, green; and *C. floridanus*, red. Clades for “antennal *IRs*” are highlighted in red. The clades for ionotropic glutamate receptors were collapsed.

Unlike *Ors* and *Gr*s, *Ir* genes have maintained relatively stable copy numbers during ant evolution (Figure 4; see Figure S8 and Dataset S4 for the full version of *IR* phylogeny with gene names and bootstrap values). While multiple duplications are likely to have occurred in the ancestor of Formicidae, unambiguous orthology among *H. saltator*, *C. floridanus*, *L. humile*, and *P. barbatus* genes has been maintained across most *IR* clades. The only lineage-specific expansion of ant *Ir* genes occurred in the *IR317* subfamily, in which the number of *C. floridanus* genes increased from 1 to 7, partially due to tandem duplications. The evolutionary history of *Ir* genes across Protostomia (e.g. nematodes, arthropods, and molluscs) has been described, where *Ir* genes are classified into “antennal *IRs*”, which are more conserved, and “divergent *IRs*”; of the seven antennal *IRs*, one (*IR21a*) was only found in *N. vitripennis* [26]. Nevertheless, orthologs of the other 6 antennal *IRs*, including *IR8a*, *IR25a*, and *IR76b* (which are thought to code for Ir co-receptors, that may play similar roles as the Orco Or coreceptor) [24], [25], as well as *IR68a*, *IR75u/f*, and *IR93a*,—were found in ants (also see Figure S9 for the *IR* phylogeny with *D. melanogaster* and *T. castaneum* sequences). In addition, there were 13 other subfamilies of divergent *IRs*. Of these divergent *IRs*, no ortholog is present in the genome of *N. vitripennis* and only one is found in *A. mellifera*, which could be due to ant specific duplications and/or preferential retention of these divergent *IRs* occurred in ants.

### Evolutionary dynamics

**Figure 5:**
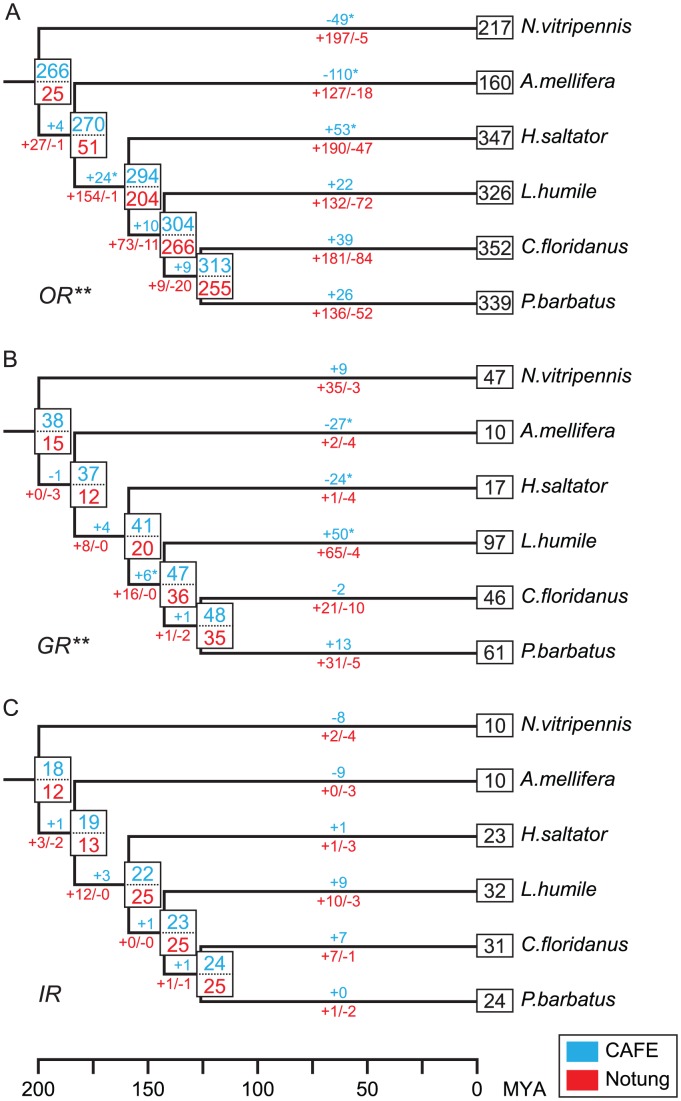
Estimated numbers of gene birth-and-death events and ancestral gene copies for chemosensory gene families. (A) *OR* family. (B) *GR* family. (C) *IR* family. The results of CAFÉ and Notung are highlighted in blue and red, respectively. Numbers above branches indicate net copy number changes estimated by CAFÉ. Numbers below branches with plus and negative signs indicate the number of gene gain and loss events estimated by Notung, respectively. Single asterisk indicates significant branch-specific expansions/contractions. Double asterisk indicates gene families that significantly violate the random gene birth and death assumption of CAFÉ. The phylogeny of Hymenoptera and the time scale are from [1], [102].

To further understand the evolutionary dynamics of chemosensory receptor genes, we quantified the gene birth and death events and estimated the number of ancestral gene copies in each family using both the maximum-likelihood (ML) and the parsimony based methods implemented in CAFÉ [62] and Notung [63], respectively. For all three families, the ML method suggested relatively high copy numbers in the ancestor of Hymenoptera (Figure 5). For instance, it estimated a repertoire of 266 *Or* genes in the hymenopteran ancestor, which was expanded in all ant lineages, but significantly contracted in both *N. vitripennis* and *A. mellifera*. A similar pattern was also observed in both the *GR* and *IR* families. Moreover, the ML analysis suggested that the low number of *Gr* genes in *H. saltator* is due to a significant gene loss in this lineage.

On the other hand, the parsimony approach gave conservative estimates of ancestral copy numbers and showed that many more gene-gain events occurred during later stages of hymenopteran evolution. According to the parsimony analysis, the number of *Or* genes increased from 25 in the last common ancestor of Hymenoptera to about 200 in *N. vitripennis* and *A. mellifera*, and more than 300 in all four ants (Figure 5A). Most notably, the repertoire of *Or* genes increased by three-fold in the ancestor of ants (from 51 to 204 copies), after the separation of *A. mellifera*, and continued to expand greatly along each ant lineage. Interestingly, although to a lesser degree, the ML method also identified significant expansion on the branch leading to the ant ancestor. In addition to the large number of gene gains, substantial gene losses also occurred in all ants. On the other hand, most duplications of ant *Grs* occurred in *C. floridanus*, *L. humile*, and *P. barbatus*, while there were only one gene gain and four gene loss events on the lineage to *H. saltator* (Figure 5B). Similar to the *OR* and *GR* families, the number of *Ir* genes also doubled in the ancestor of ants after its separation from other Hymenoptera (Figure 5C). Subsequent increase of *Ir* gene number was only observed in *C. floridanus* and *L. humile*.

Overall, the ML and parsimony analyses gave different estimates of the ancestral copy numbers and gene gain and loss events. The ML method assumes a random gene birth and death process [64], which is significantly violated by both the *OR* and *GR* families (p-values<0.01). On the other hand, the parsimony approach aims to minimize the number of gene gain and loss events, and thus might underestimate the number of ancestral copies. Nonetheless, both analyses support the hypothesis that chemosensory genes have distinct evolutionary dynamics in ant lineages in comparison to the other two hymenopterans.

### Antennal expression profiles of ant chemosensory receptor genes

In insects, most *Ors* and some *Grs*/*Irs* are expressed in antennal ORNs [18], [24], [49], [65]. As best illustrated in studies of the *Drosophila* olfactory system, each ORN expresses a single tuning Or which is responsible for the odorant response profile and all the ORNs expressing that singular tuning Or send axonal connections to a single antennal lobe glomerulus thereby providing a mechanistic basis for the initial stages of odor coding [18]. Therefore, we analyzed antennal transcriptomes of workers and males for both *C. floridanus* and *H. saltator*, to identify chemosensory receptor genes that are differentially expressed between castes (minors and majors in *C. floridanus*) and between different sexes, and which might play salient roles in social communication (see Table S2 for information on transcriptome datasets).

We performed pairwise comparisons between males and females within *C. floridanus* and *H. saltator* (Dataset S5). At the whole transcriptome level, there was a very high similarity between major and minor worker of *C. floridanus* (r^2^ = 0.99; Figure S10A), while greater diversity was found between workers and males (r^2^ values around 0.85 for all comparisons), largely due to mild up-regulation of many genes in males (Figure S10B, S10C). Similar trends were also observed for chemosensory receptor genes (Figure S10D).

#### *OR* family

In both sexes of *C. floridanus* and *H. saltator*, the ortholog of *Orco* was consistently the most highly expressed *Or* gene. It accounted for ∼15%–20% of all the *Or* gene expression in *C. floridanus* and ∼6%–8% in *H. saltator*. For the repertoire of tuning *Ors* within each species, almost all of them were expressed in workers at levels above the medians of their respective antennal transcriptomes (which was used as the criterion for expression versus non-expression of chemosensory gene in the present study). In contrast, only one third of the tuning *Or* genes were expressed in males of both ants. These comparisons identified almost 40 *Ors* in *C. floridanus* and 120 *Ors* in *H. saltator* that displayed significant differential expression between workers and males (Table 1, Dataset S5). Interestingly, ∼95% of these genes were enriched in workers, almost all of which had below-median expression levels in males. In addition, we found 13 *Or* genes that were differentially expressed between major and minor workers of *C. floridanus* (Table 1, Dataset S5). However, the log2 fold-changes of these genes (less than 1.5) were much lower than those of the genes (greater than 3) revealed in worker vs. male comparisons.

**Table 1:** Significantly differentially expressed *C. floridanus* and *H. saltator* chemosensory receptor genes revealed by analysis of antennal transcriptomes.

Species	Comparison	*OR*	*GR*	*IR*
*C. floridanus*	Major worker vs. Male	38 (1)	0 (0)	1 (1)
	Minor worker vs. Male	42 (1)	0 (0)	1 (1)
	Major vs. minor worker	13 (0)	2 (1)	0 (0)
*H. saltator*	Worker vs. Male	120 (4)	1 (0)	2 (2)

Significantly differentially expressed genes were identified by using Cuffdiff (q-value≤0.05). Number in bracket indicates the number of genes with higher expression level in male (or minor worker in the major worker vs. minor worker comparison).

**Figure 6:**
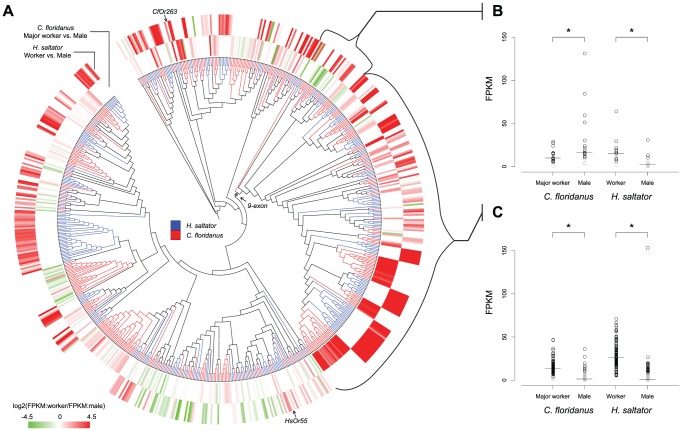
Diversified expressions of evolutionarily related *Or* genes. (A) Expression profiles of *C. floridanus* and *H. saltator Ors* shown along with the phylogeny of *Or* genes. In the phylogenetic tree, *C. floridanus Ors* are labeled by red and *H. saltator Ors* by blue. In the heat-map, red color indicates higher expression level in worker and green indicates higher expression level in male. The inner circle shows the relative expressions of *C. floridanus Ors* between major worker and male (comparison between minor worker and male not shown because of the highly similar expression profiles of *C. floridanus* major and minor workers); the outer circle shows the relative expressions of *H. saltator Ors* between worker and male. FPKM stands for Fragments Per Kilobase of exon per Million fragments mapped. (B) Expression levels of *Ors* belonging to the three basal clades in the 9-exon subfamilies. *C. floridanus Ors* had significantly higher expression in male (*p*-value<0.05; Wilcoxon ranked-sum test), while *H. saltator Ors* had significantly higher expressions in worker (*p*-value<1e-4; Wilcoxon ranked-sum test). (C) Expression levels of the remaining *Ors* in the 9-exon subfamilies. For both *C. floridanus* and *H. saltator*, *Ors* had significantly higher expressions in worker (*p*-value<1e-15; Wilcoxon ranked-sum test). Short lines indicate median expression levels for each gene set. For both panels (B) and (C), genes expressed below the medians of their respective transcriptomes were labeled by grey.

To investigate the relationship between evolutionary relatedness and expression regulation of *Or* genes, we mapped results of worker vs. male comparisons to the phylogeny of *C. floridanus* and *H. saltator Or* genes. As shown in Figure 6A, there are multiple examples where *Or* genes in one ant species showed sex-specific-enrichment patterns similar (or opposite) to closely related homologs in the other ant species. Notably, the *9-exon Or* subfamily illustrates both situations described above (Figure 6B, 6C). In the three basal clades, *C. floridanus* genes were mostly enriched in male, while all but one *H. saltator* gene had higher expression levels in workers (Figure 6B). In contrast, all the remaining *Or* genes formed a well-supported monophyletic clade and almost all of them were enriched in workers for both *C. floridanus* and *H. saltator* (Figure 6C). We further examined the expression patterns of (co-)orthologous genes in the two ant species. Using bootstrap values of 70 as threshold, we delineated 98 orthologous groups of *C. floridanus* and *H. saltator Or* genes, of which 41 groups included at least one gene from each ant species being differentially expressed by at least two-fold between males and females. *C. floridanus* and *H. saltator* genes were enriched in the same sex in 29 of the 41 groups and in different sexes in other 10 groups (Table S3). The remaining 2 groups showed conflicting expression patterns within species.

#### *GR* family

**Figure 7:**
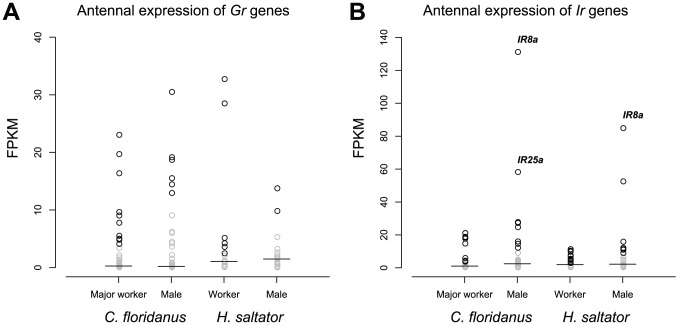
Expression levels of *Gr* and *Ir* genes. (A) The *GR* family. (B) The *IR* family. Short lines indicate median expression levels for each gene set. In both panels, genes expressed below the medians of their respective transcriptomes were labeled by grey.

Unlike *Or* genes, only a portion of *Gr* genes within each species were expressed in workers (less than 25% for *C. floridanus* and ∼35% for *H. saltator*) and males (less than 15% for both ants) (Figure 7A). Furthermore, worker vs. male comparisons revealed only one *H. saltator Gr* gene that was differentially expressed between worker and male (*HsGr*7), and none in *C. floridanus*. While two *C. floridanus Gr* genes were found to have differential expressions between major and minor workers (*CfGr9* and *CfGr54*), their absolute expression values were close to or below the median of their respective transcriptomes.

#### *IR* family

50% or less of *Ir* genes of each species were expressed in any given sex (Figure 7B), and almost all expressed *Ir* genes are conserved “antennal *IRs*”. We identified only one *C. floridanus Ir* gene and two *H. saltator Ir* genes that have differential expressions between workers and males. Interestingly, all of these *Ir* genes were enriched in male. The ortholog of *IR8a*, encoding one of the Ir co-receptors [24], [25], was differentially expressed in both *C. floridanus* and *H. saltator*, and was also the most highly expressed *Ir* gene in males of both ants, while another “antennal *IR*” (*HsIR75u.2*) was also found to be more highly expressed in *H. saltator* males than in workers.

### Identification of a ligand for a differentially expressed ant odorant receptor

**Figure 8:**
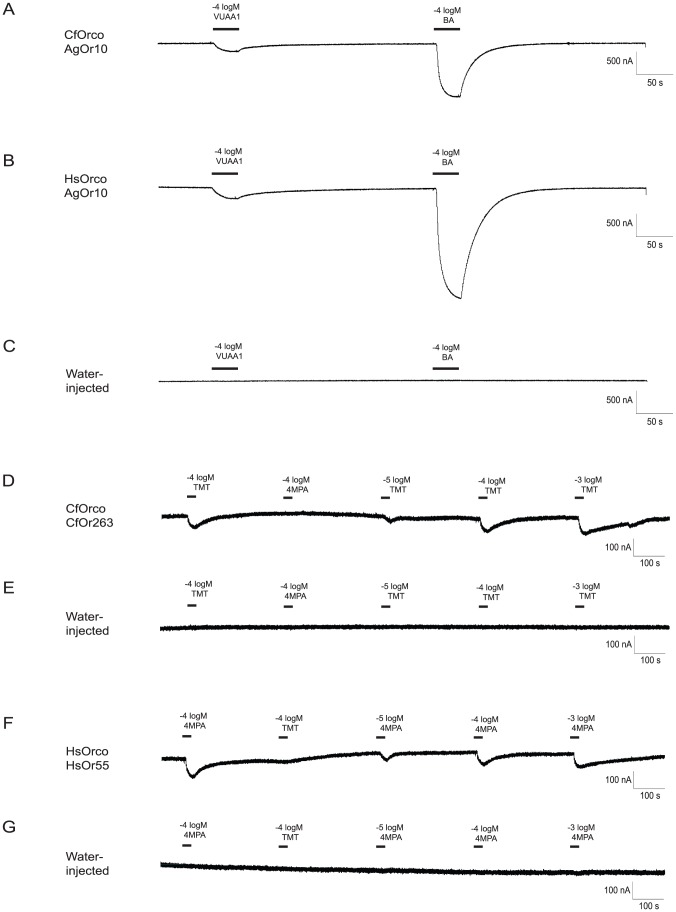
Identification of ligands for *C. floridanus* and *H. saltator* Ors. When paired with a previously characterized mosquito Or (AgOR10) [103], both CfOrco (*C. floridanus* Orco) (A) and HsOrco (*H.saltator* Orco) (B) produced responses to VUAA1 (an agonist for Orco) [35] and benzaldehyde (BA, an agonist for AgOR10) in *Xenopus* oocytes. These responses were not observed in water-injected control oocytes (C). The novel *C. floridanus* tuning Or CfOr263 also shows a specific and dose-dependent response to 2,4,5-trimethylthiazole (TMT) (D), while the novel *H. saltator* tuning Or HsOr55 shows a similar dose-dependent response to 4-methoxyphenylacetone (4 MPA) (F). Neither response is observed in water-injected oocytes (E,G).

In order to validate our bioinformatic annotations and in an attempt to link functional data to the antennal expression data, we have cloned a small subset of 14 *C. floridanus* and *H. saltator Or* genes, drawn from 6 subfamilies in the *Or* phylogeny (*D*, *E*, *H*, *L*, *V*, and *9-exon*). These include four genes (*CfOr263*, *HsOr212*, *HsOr213*, and *HsOr279*) that display significant differential expression in our transcriptome analysis (see Methods and Materials for full list). This allowed us to carry out deorphanization studies to decipher the odorant response profiles of these receptors through the use of two-electrode voltage clamp recordings in *Xenopus* oocytes heterologously expressing ant Ors [44], [66]. After first confirming that the *C. floridanus* and *H. saltator* Orco proteins showed coreceptor function in combination with a previously deorphanized mosquito tuning Or (Figure 8A, 8B), candidate ant tuning Ors were screened against a panel of 73 unitary and complex stimuli (Table S4). These stimuli consisted of a variety of general odorants, as well as hydrocarbons known to be produced by *H. saltator* or *C. floridanus*.

Out of the 14 tuning Ors initially screened, CfOr263 (from *OR* subfamily *D*; Figure 2), which is highly expressed in workers as compared to males (Figure 6A), produced specific and dose-dependent responses to 2,4,5-trimethylthiazole (Figure 8D, 8F), a naturally occurring odorant found in cooked beef and pork [67] found in the library of general odorants that we screened. An additional Or from *H. saltator*, HsOr55 (from *OR* subfamily *L*; Figure 2), showed a dose-dependent response to another odorant from our general odorant library, 4-methoxyphenylacetone (Figure 8E, 8G), which is a naturally occurring odorant found in anise essential oil [68]. However, this particular Or has not been shown to be differentially expressed between males and females. It should also be noted that, as is the case for most ant Ors, both receptors have multiple closely related homologs that may possess similar chemosensory functions (Figure 6A).

## Discussion

### Expanded ant chemosensory receptor repertoire

We have developed and used a dedicated annotation scheme to comprehensively elucidate the repertoire of chemosensory receptor genes in both *C. floridanus* and *H. saltator*. Through exhaustive homology search and careful manual curation, we significantly improved upon previous studies to identify roughly equivalent numbers of *Or*/*Gr*/*Ir* genes in the genomes of *C. floridanus* and *H. saltator* as compared to two other sequenced ant genomes [47], [48], providing a solid foundation for subsequent study.

It is striking that, in general, ants have the most expanded repertoire of chemosensory receptor genes in Hymenoptera (Figure 1). The numbers of ant *OR* and *IR* family members are much greater than those of the other two hymenopteran genomes currently available. Indeed, thus far, ant genomes have the largest number of *Or* genes among all insects [69]. Furthermore, although the number of the *Gr* genes varies greatly among hymenopterans and also within ants, *L. humile* carries the largest *Gr* family; it has about 2- and 10-fold more *Grs* than *N. vitripennis* and *A. mellifera*, respectively. Interestingly, although ants and honey bees are both social insects, ants have much larger repertoires of all three chemosensory receptor gene families than honey bees, possibly indicative of a more sophisticated communication system relying on chemicals [70].

Our phylogenetic analyses of hymenopteran chemosensory receptor genes reveal distinct evolutionary patterns among gene families. Among chemosensory receptors, the *OR* family shows the most dramatic birth-and-death evolution, with many *OR* subfamilies displaying diversified patterns of gene gain-and-loss. For example, the *9-exon* subfamily and others have experienced rapid gene duplications at almost all stages of Hymenoptera evolution, followed by numerous losses of duplicates. In contrast, there are 35 subclades that have only one ortholog in all four ants. Further, the *IR* family has maintained relatively stable copy numbers in ants; lineage-specific expansion only occurred in *C. floridanus* and *L. humile* for two of the 13 “divergent *IRs*”. In between these extremes is the *GR* family that has expanded moderately in *N. vitripennis* and three of the four ants.

Recent studies of chemosensory receptors in mammals and *Drosophila*, as well as other genes with important regulatory and physiological functions, have suggested a possible correlation between functional requirements and the variations of gene numbers [52], [71], [72]. Genes with conserved roles tend to have relatively stable copy numbers while those with diversified functions have higher rates of birth-and-death, although the degrees of copy number changes are somewhat random. Our results suggest that this pattern could also hold true for the evolution of the hymenopteran chemosensory receptor genes. For example, as an obligatory co-receptor for all other Ors [29], *Orco* is the most conserved insect *Or* gene and also the only one that has maintained unambiguous orthology in all insects studied to date, including ants [69]. Similarly, orthologs of most “antennal *IRs*” [26] have also maintained strict single-copy in Hymenoptera. It has been proposed that these conserved “antennal IRs” represent the earliest insect chemosensory receptors and perform functions important for all insects [26]. Therefore, we suggest that the chemosensory receptor genes that have constant copy numbers in ants (e.g. the 35 single-copy tuning *Ors*) are likely to carry out important functions common for all ants.

On the other hand, prevalent rapid expansions in chemosensory receptor gene families could allow for diversification in ligand specificity/sensitivity among duplicated receptor genes. Such functional divergences would offer tremendous opportunities for organisms to explore different chemical niches, thus facilitating the adaption to new environments and/or the evolution of novel life styles such as sociality. In all three gene families, we found either retention of the complete ancestral repertoire (according to the ML method) or dramatic increases in gene numbers (according to the parsimony method) in the ancestor of ants (Figure 5), which might have contributed to the success and subsequent diversification of this group.

In addition, there are many cases of unbalanced expansions/contractions among lineages in specific (sub-)families, suggesting that the chemosensory receptor repertoire has been differentially exploited among ants, which might shed light on the evolution of different lifestyles of ants. For example, our results indicate expansions of *Grs* in *C. floridanus*, *L. humile*, and *P. barbatus*, but not *H. saltator*, which are likely to reflect differences in their feeding behaviors. In this view, scavengers like *C. floridanus* might require a highly expanded repertoire of taste receptors to discriminate nutritious food sources from spoiled, contaminated, or poisoned substrates. In contrast, *H. saltator* workers likely rely more on visual cues to track down prey, as suggested by their large eyes and expanded number of ommatidia [73]. Furthermore, Grs which act as contact chemoreceptors would be far less useful for identifying and capturing prey. In fact, ponerine ants in general rarely use liquid food sources, since they normally lack the ability to exchange liquids stored in their crop [74] which further reduces the potential benefit of a large Gr repertoire.

Another intriguing possibility is that Grs are involved in the contact chemosensation of species-specific, nonvolatile CHCs (e.g. queen pheromone, nestmate recognition signals, etc.), and that *C. floridanus* has more Grs precisely because they utilize a greater number and variety of pheromones to support their more rigid and complex social lifestyle. Presumably, these Grs would be in addition to the large number of worker enhanced *Ors* that are likely to be involved in the same process. Furthermore, *C. floridanus* has expansions in multiple *GR* subfamilies, including 5 homologs of the *DmGr43a*/*AmGr3* gene, which has been recently shown to be a fructose receptor [75]. Taken together, our results indicate a correlation between the expanded *GR* family and the more complex chemical ecology of *C. floridanus*.

### Diversified expression of chemosensory receptor gene

The antenna is perhaps the most important chemosensory organ for ants, where a variety of ant species have been observed to closely inspect their environment and each other by touching their antennae in a process known as antennation [2]. This makes it likely that most of the behaviorally important chemosensory neurons (and their corresponding chemosensory receptors) are located in this organ. Our comparative analysis of antennal transcriptomes of workers and males in both *C. floridanus* and *H. saltator* reveal differential expressions of chemosensory receptor genes both within and between species, providing important clues on their functional divergence.

One major pattern revealed by our results is the substantial sexual dimorphism in chemosensory receptor gene expression in ants. For both *C. floridanus* and *H. saltator*, almost all *Ors* were expressed in workers, but only one third were expressed in male. Similarly, workers consistently had more expressed *Grs* and *Irs* than males. In contrast, expression of chemosensory receptor genes was highly similar between major and minor workers in *C. floridanus*. Previous studies have shown that the antennal lobes of males from both *C. floridanus* and *H. saltator* lack a large subset of glomeruli relative to workers [76], [77], [78], which may explain the low number of chemosensory receptor genes expressed in males. Given that the number of glomeruli in insects generally correlates with the number of functional odorant receptors [18], [65], it is likely that most of the *Ors* that are only expressed in *C. floridanus* and *H. saltator* workers project to these female-specific glomeruli. Furthermore, it has been shown in another *Camponotus* species (*Camponotus japanicus*) that females exclusively possess the olfactory sensilla necessary to detect non-nestmate CHCs, [79], [80]. It is therefore likely that the CHCs receptors are encoded by some of the worker-specific *Ors* in *C. floridanus*. In particular, the *9-exon* subfamily represents the largest expansion of *Ors* in all ants and it harbors close to 100 worker-specific *Ors* in both *C. floridanus* and *H. saltator*. These results strongly support previous hypothesis that members of the *9-exon* subfamily are likely candidates for ant CHCs receptors [47], [48]. These *Ors* are potentially involved in detecting CHCs involved in worker-to-worker or worker-to-queen intracolonial social communication.

Interestingly, we also noticed discrepancies between the overall number of *Ors* and the number of glomeruli in the adults of these two ant species. *H. saltator* workers and males both have far more expressed *Ors* than the number of glomeruli in the adult antennal lobe (approximately 78 in the adult male and 178 in the adult worker [77]). The discrepancy in *H. saltator* could possibly be the result of co-expression of multiple tuning Ors in the same ORN and/or the projection of ORNs expressing different, but related tuning Ors to the same glomerulus, which have both been observed for a small number of Ors/ORNs in *D. melanogaster* [81], [82], [83], [84].

However, given that the number of expressed *Ors* is about twice the number of observed glomeruli, this would mean that each glomerulus received input from, on average, two odorant receptors. Although co-expression of tuning Ors has not been observed to such a broad extent in any insect olfactory system studied to date, it should be noted that many of the receptor pairs that are co-expressed in *Drosophila* appear to be the result of tandem duplication events [84]. Therefore, it is possible that the extensive tandem duplication of *H. saltator Or* genes may also result in the co-expression of closely related odorant receptors from the same clusters. All of these are highly interesting hypotheses that may be examined in future studies.

In contrast to *H. saltator*, *C. floridanus* has approximately 80 fewer *Ors* than the number of adult worker glomeruli (about 454 [76]). In this instance it is possible that many of those glomeruli receive projections from *Gr* and *Ir* expressing ORNs, as there is precedence for this in *Drosophila* [24], [43] and the number of predicted *Grs* and *Irs* would be enough to fill the gap. Moreover, it could be that several *Ors* have been missed by the current analysis due to incomplete genome assembly; some of the fragmented *Or* gene models might represent genuine genes, and further genomic/transcriptomic data would help address this possibility.

Although chemosensory receptor genes in general had higher expression in workers, our studies have nevertheless identified a single *Or* (*CfOr267*, in subfamily *9-exon*) and a single *Ir* (*CfIR8a*) in *C. floridanus*, as well as 4 *Ors* (*HsOr32*, *HsOr35*, and *HsOr37*, in subfamily L; and *HsOr224*, in subfamily *E*) and 2 *Irs* (*HsIR8a* and *HsIR75u.2*) in *H. saltator* that were significantly male-enriched. The male-enrichment of a receptor gene could be due to elevated expression of the gene in ORNs of males relative to workers, and/or increased number of ORNs expressing the gene in males. No matter which of the possibilities is indeed the case, our results indicate higher overall abundances of these chemosensory receptor genes in male antennae. These genes are viable candidates for receptors that are specifically tuned for male-specific social cues, including queen pheromones. In fact, at least one male-specific honeybee odorant receptor that responds to a queen-specific pheromone has already been revealed through microarray analysis and subsequent functional characterization in *Xenopus* oocytes [85]. It would not be surprising to see that similar results will be found with the male-enriched ant *Ors*.

In insects, the co-receptors *IR8a* and *IR25a* are the two most conserved *Irs* [26]. Although a systematic profiling of sexual dimorphic *Ir* expression is still lacking, a previous study has shown that the *Anopheles gambiae* orthologs of both *IR8a* and *IR25a* have higher expression in female than male [49]. Interestingly, *IR8a* was the most male-enriched *Ir* in both *C. floridanus* and *H. saltator*. While *IR25a* also displayed higher expression in *C. floridanus* male, it was not expressed in the male of *H. saltator*. These results could possibly indicate a functional divergence of *IR8a* and *IR25a* between Diptera and Hymenoptera. In addition, the high expression of *IR25a* in males of *C. floridanus,* but not *H. saltator*, suggests that IR25a-mediated signaling might have contributed to the more expanded roles for males within the colony of the former species. It may be that *C. floridanus* males are more involved in intracolonial interactions than *H. saltator* males, since males from other *Camponotus* species are known to participate in food exchange in the colony [86], which has not observed in *H. saltator* males.

We have also found diversified expression of closely related *Ors* within and between species. For example, in the basal clades of the *9-exon OR* subfamily, closely related *C. floridanus* and *H. saltator Ors* showed opposite sexual dimorphism in their expression (Figure 6B). Although the well-supported monophyletic clade within the *9-exon OR* subfamily mostly consists of worker-enriched genes, it also harbors a few genes that are highly enriched in male (Figure 6C). Thus, while our expression results are generally (and strongly) consistent with the idea that members of the *9-exon OR* subfamily are involved in the detection of CHCs by workers [47], a subset of these receptors have apparently been adapted for use in males, possibly for detecting queen mating pheromones.

Taken together, these results indicate that ant *Or* genes have experienced not only extensive gain-and-loss, but also rapid changes in their expression, once again highlighting the highly dynamic nature of chemosensory receptor gene evolution. Our phylogenetic and transcriptomic analyses, in combination, have identified ant chemosensory receptor genes that exhibit evolutionary and expression patterns indicative of species/sex-specific functions. Ultimately, deorphanization of these receptors will greatly facilitate our understanding of the chemical ecology of social lifestyle in ants.

### Heterologous characterization of differentially expressed *C. floridanus Ors*

In our heterologous studies of ant tuning Ors, we have identified chemical agonists for a single receptor from each of the two species analyzed. These data provide conclusive validations for our bioinformatic-based annotations. Although a honeybee odorant receptor has been previously shown to respond to the queen substance 9-oxo-2-decenoic acid [85], we believe that this represents the first published report of ligand activators for odorant receptors from ants.

In these studies, HsOr55 from *H. saltator*, display significant responses to 4-methoxyphenylacetone, a naturally occurring odorant found in anise essential oil [68]. Since anise essential oil has been shown to have a repellent and/or insecticidal effect on at least some species of insects [87], [88], 4-methoxyphenylacetone might represent a general insect repellent, with HsOr55 acting as the detector for this repellent in *H. saltator*. Whatever HsOr55's role may be, it is likely to be a very general one, since *HsOr55* transcripts do not appear to be differentially expressed between workers and males.

The other odorant receptor characterized in this study, CfOr263 from *C. floridanus*, displayed sensitivity to 2,4,5-trimethylethiazole, a naturally occurring odorant found in cooked beef and pork [67] that has been previously shown to induce strong responses in the CpC neuron of the maxillary palp in the mosquito *Anopheles gambiae* [44]. While the relevance of this chemical to *C. floridanus* remains unclear, the fact that *CfOr263* transcripts are enriched in workers relative to males suggests that this odorant may be an important volatile semiochemical for *C. floridanus* workers. Regardless, the successful identification of odors that activate CfOr263 and HsOr55 strongly validates the role of ant Ors as chemosensory receptors. Furthermore, the large differential expression of *CfOr263* between workers and males indicates that it is detecting a sex- specific signal that is relevant to workers but not to males, and testing a broader panel of odorants in the future will provide a better understanding of what that signal might be.

### Conclusions

We have revealed a greatly expanded repertoire of chemosensory receptor genes for a pair of divergent ant species, including about 400 *Ors* and an order of magnitude smaller number of *Grs* and *Irs*. Phylogenetic analysis of these newly annotated genes indicates that there are likely to be vast differences in the importance of particular chemoreceptor families and subfamilies between the four ant species examined, which is likely to reflect the variety of ecological and social demands experienced the members of each species. These analyses also reveal high rates of gene birth-and-death evolution among the olfactory and gustatory receptor genes, suggesting that some factor (such as changes in the complex CHC profiles that control ant social behavior) is driving rapid evolution in their chemical response profiles. The large repertoire of ant chemosensory genes might be either due to preferential retention of ancestral genes or rapid expansions in the ant ancestor and during later stages of ant evolution. To further complement these phylogenetic results, we have generated and analyzed antennal-specific RNAseq expression data to identify ∼40 *C. floridanus* and ∼120 *H. saltator* chemosensory receptors that exhibit significant sexual dimorphism in expression. This expression data has, in turn, informed studies towards the identification of odorant ligands for socially relevant receptors, a process that we have already successfully accomplished in a heterologous system for one of the differentially expressed *C. floridanus Ors*. Taken together, our evolutionary analysis, transcriptome profiling, and heterologous characterization provide new insights into the roles of the chemosensory receptors in inter-sex behavioral and social differences of ants.

## Materials and Methods

### Gene annotations

The assemblies of *C. floridanus* (version 3.5) and *H. saltator* (version 3.5) were downloaded from the Hymenoptera Genome Database [89]. Protein sequences of reported chemosensory gene were also collected from *Apis mellifera*, *Acyrthosiphon pisum*, *Drosophila melanogaster*, *Nasonia vitripennis*, *L. humile,* and *P. barbatus* [15], [26], [28], [47], [48], [50], [54]. An in-house bioinformatics pipeline was developed to identify candidate chemosensory genes in *C. floridanus* and *H. saltator*. First, all collected chemosensory gene sequences were searched against the two ant genomes using TBLASTN [90] with an e-value cutoff of 1e-5. Resulting High-scoring Segment Pairs (HSPs) were sorted by their blast bit-scores, and an average bit-score of the top 75% HSPs were calculated. Any HSPs with a bit-score less than 25% of the average was discarded. Chains of HSPs were than created from retained HSPs. Two HSPs were chained together if the following criteria were met: 1) they are derived from the same query; 2) they are located within 3 kb on the same strand of a scaffold/contig; and 3) the corresponding query region of the upstream HSPs must also be N-terminal to that of the downstream HSPs. The third criterion was applied to avoid artificial concatenation of neighboring chemosensory genes. Genomic regions covered by HSPs chains were considered putative chemosensory gene coding regions. For each putative gene, we then selected the query corresponding to the highest scoring HSPs at that region as reference sequence for homology-based gene prediction using GeneWise (version 2.2.0) [91]. All predictions were sorted by ORF length and the lowest 25% was filtered. This pipeline was iterated by adding results of previous run to input until no additional genes were found.

Multiple sequence alignments (MSAs) of predicted OR/GR/IRs were constructed using MUSCLE (version 3.8) [92] and manually inspected. Attempts to improve annotations were made whenever an obvious problem was identified (e.g. missing exon, incorrect exon-exon junction). In addition, in the *OR* and *GR* families, we observed many fragmented gene models, likely due to pseudogenization and incomplete genome assembly. For the convenience of subsequent analyses, a minimum size cutoff of 300 amino acids was used for the ORs and GRs. For IRs, we screened all predicted protein sequences with InterProScan (V4.8) [93] and filtered the ones without characteristic domains of IR (PF10613 and PF00060) [26].

### Phylogenetic analysis

We included in our phylogenetic analysis chemosensory receptor genes in six hymenopteran species, including *A. mellifera*, *C. floridanus, H. saltator, N. vitripennis*, *L. humile,* and *P. barbatus*. For each of the *OR*/*GR*/*IR* families, all family members were firstly aligned at once using MUSCLE (version 3.8) and a preliminary phylogenetic tree was built using RAxML (version 7.2.8) [94]. Sequences were then divided into groups corresponding to highly supported clades in the preliminary phylogeny. Groups were aligned individually using PROBALIGN (version 1.4) [95] and then combined together using the profile alignment function of MUSCLE. The complete alignment were further manually inspected and adjusted using GeneDoc (version 2.6) [96]. In addition, poorly aligned regions in the alignment were removed using trimAl (version 1.4) [97]. The final maximum-likelihood tree was constructed using RAxML with Le-Gascuel (LG) substitution model [98] and GAMMA correction for rate variation among sites. Reliability of tree topology was evaluated by 100 bootstrap replicates. To estimate the number of gene gain and loss events, we used a maximum-likelihood based approach implemented in CAFÉ (version 2.2) [62] with default settings. As an alternative approach, we also used the parsimony based “modified reconciliation method” [99]; we first collapsed branches with bootstrap support lower than 70 in phylogenies of *OR*/*GR*/*IR* families and then reconciled condensed trees with known organismal relationships using Notung (version 2.6) [63].

### Antenna collection, RNA extraction, and Illumina sequencing

Samples originated from *C. floridanus* colonies that had been founded in the Liebig lab from queens captured in southern Florida between 2002 and 2009 and from *H. saltator* colonies collected in Karnataka, India between 1995 and 1999. Antennae were collected from each of five groups of adult ants: *H. saltator* workers and males and *C. floridanus* major workers, minor workers, and males. Whole ants were flash-frozen in liquid nitrogen and kept on dry ice as 100 antennae from each group were removed with forceps. Antennae were placed directly into RNAlater ICE (Ambion) that had been pre-chilled on dry ice in a conical, ground-glass, tissue homogenizer. RNAlater ICE was replaced with 1 ml Trizol (Invitrogen), in which antennae were homogenized. Total RNA was isolated following Trizol manufacturer instructions; briefly, after addition of 200 µl of a chloroform∶isoamylalcohol mixture (24∶1), each sample was mixed vigorously and the RNA-containing aqueous layer was isolated with centrifugation. RNA was further purified and DNAse-treated with the RNeasy Miniprep kit (Qiagen). After ethanol-precipitation, the RNA pellet was resuspended in 30 µl nuclease-free water. Male samples were sequenced using Illumina HiSeq2000 at the NYULMC Genome Technology Center, generating ∼33 million 50 bp single-end reads for *C. floridanus* male and ∼164 million 51 bp single-end reads for *H. saltator* male. All worker samples were sequenced at Hudson Alpha, generating more than 20 million 50 bp paired-end reads for each sample (sum of two technical replicates).

### Analysis of ant antennal transcriptome

Reads of *C. floridanus* male sample were trimmed to 34 bp (8 bp trimmed from both ends) to remove low-quality positions. In addition, for all worker datasets, we treated each paired-end read as two single-end reads. Therefore, all datasets in our subsequent analyses consist of only single-end reads. Alternative strategies for data processing led to highly similar estimations of gene expression values (Table S5). For each dataset, reads were mapped to the corresponding ant genome using TopHat (version 1.3.3) [100] with default setting. Gene annotations for *C. floridanus* (version 3.5) and *H. saltator* (version 3.5) were downloaded from the Hymenoptera Genome Database and used in combination with our annotation of chemosensory genes to guide the reads mapping. Gene expression levels (in FPKM values) and differentially expressed genes were determined using Cuffdiff v1.3.0 [101] with frag-bias-correct, multi-read-correct, and upper-quartile-norm options turned on.

### Heterologous analysis of ant odorant receptors

Predicted *Or* coding sequences were amplified, by PCR, from *H. saltator* and *C. floridanus* worker antennal cDNA samples obtained from colonies established at Arizona State University (Tempe, AZ). The PCR-amplified sequences were then TOPO cloned into the Gateway Entry vector pENTR/D-TOPO (Life Technologies), followed by an additional cloning step into a destination vector derived from pSP64T. To obtain cRNA for each *Or*, the pSP64T vector containing the appropriate coding sequence was linearized by restriction digest and used as a template for cRNA synthesis using the mMessage mMachine Sp6 Kit (Ambion). Heterologous expression of ORs was accomplished as described previously [66]. Briefly, mature oocytes were surgically extracted from *Xenopus leavis* adult females, treated with 2 mg/mL collagenase II in 1× Ringer's solution (96 mM NaCl, 2 mM KCl, 5 mM MgCl_2_, and 5 mM Hepes, pH 7.6) for 30–45 minutes at room temperature, and then injected with 27.6 nL of a 1∶1 mixture (by mass) of a given tuning *Or* in combination with the appropriate *Orco* ortholog (either *HsOrco* or *CfOrco*). After injection, oocytes were stored in Incubation Medium (10% dialyzed horse serum in 1× Ringer's solution) at 18C for 3–7 days before testing. Responses to odorants were measured by recording whole-cell currents in Clampex 10.2 (Molecular Devices) using a two-electrode voltage-clamp setup (OC-725C, Warner Instruments) maintained at a −80 mV holding potential. Odorants were first dissolved in DMSO, and then further diluted into Ringer's solution before being introduced to the oocyte recording chamber using a perfusion system. For the hydrocarbons that were tested, 0.01% Triton X-100 (Sigma) was also added to the Ringer's solution to aid in dissolving the odorant. The following odorant receptors were tested with the odorants listed in Table S4: CfOr183, CfOr215, CfOr263, HsOr19, HsOr55, HsOr132, HsOr170, HsOr175, HsOr212, HsOr213, HsOr234, HsOr239, HsOr279, HsOr287.

### Chemicals

Odorant chemicals were purchased from commercial sources at the highest purity available. Henkel 100, a mixture of 100 different volatile chemicals, was obtained from Henkel (Düsseldorf, Germany), and the C7–C40 saturated alkane mixture was purchased from Supelco (Bellefonte, PA, USA).

## Supporting Information

Dataset S1 Details of *C. floridanus* and *H. saltator* chemosensory receptor genes annotated in this study. The genome location and predicted protein and transcript sequences are provided for each annotated gene.
(XLSX)

Dataset S2 Phylogenetic relationships of Hymenoptera *Or* genes shown in newick format. Bootstrap values are shown for all nodes. See Figure 2A and Figure S2 for graphical presentation of the same phylogeny.
(TXT)

Dataset S3 Phylogenetic relationships of Hymenoptera *Gr* genes shown in newick format. Bootstrap values are shown for all nodes. See Figure 3 and Figure S6 for graphical presentation of the same phylogeny.
(TXT)

Dataset S4 Phylogenetic relationships of Hymenoptera *Ir* genes shown in newick format. Bootstrap values are shown for all nodes. See Figure 4 and Figure S8 for graphical presentation of the same phylogeny.
(TXT)

Dataset S5 Complete results of antennal transcriptome comparisons for all chemosensory receptor genes. Four pairwise comparisons are presented, including major worker vs. minor worker (*C. floridanus*), major worker vs. male (*C. floridanus*), minor worker vs. male (*C. floridanus*), and worker vs. male (*H. saltator*).
(XLSX)

Figure S1 *C. floridanus* and *H. saltator* OR genes are mostly distributed in tandemly arrayed gene clusters. (A) *C. floridanus* OR genes. (B) *H. saltator* OR genes.
(EPS)

Figure S2 Phylogenetic relationships of Hymenoptera *Or* genes. The same tree as in Figure 2A is shown with gene names. Only bootstrap values ≥50 are shown. Supported subfamilies are indicated by brackets.
(EPS)

Figure S3 Phylogenetic relationships of *Or* genes in representative insects. A maximum-likelihood tree of *Or* genes from *D. melanogaster*, *T. castaneum*, and six hymenopteran species. The topology is estimated by using RAxML with Le-Gascuel (LG) model. Reliability of internal nodes was evaluated by 100 bootstrap replicates. Only bootstrap values ≥50 are shown. Subfamilies that are delineated based on hymenoptera *OR* phylogeny are indicated by brackets. All *D. melanogaster* and *T. castaneum* genes are highlighted in blue. Confidently resolved relationships among hymenopteran and non-hymenopteran *Or* genes are indicated by red.
(EPS)

Figure S4 Phylogeny of selected OR clades exhibiting distinct modes of gene birth-and-death: (A) constant single-copy in all ants; (B) gene gain in *P. barbatus* only; (C) gene loss in *H. saltator*, but multiple gene gains in other ants; and (D) lineage-specific expansions in all ants.
(EPS)

Figure S5 Tandemly arrayed ant OR genes were generated by duplications at multiple stages of ant evolution. Evolutionary relationships and genomic arrangements of selected *C. floridanus* and *H. saltator* OR genes were shown. M, N, O, and P indicate four well supported OR subfamilies, each likely representing one OR gene in the ancestor of Hymenoptera. *C. floridanus* OR genes belonging to the cluster on scaffold538 were labeled by red. *H. saltator* OR genes belonging to the cluster on scaffold105 were labeled by blue.
(EPS)

Figure S6 Phylogenetic relationships of Hymenoptera *Gr* genes. The same tree as in Figure 3 is shown with gene names. Only bootstrap values ≥50 are shown. Supported subfamilies are indicated by brackets. Subfamilies showing interesting evolutionary patterns are named after the orthologs in *N. vitripennis* and *A. mellifera*. The other subfamilies are named as *A*–*H*.
(EPS)

Figure S7 Phylogenetic relationships of *Gr* genes in representative insects. A maximum-likelihood tree of *Gr* genes from *D. melanogaster*, *T. castaneum*, and six hymenopteran species. The topology is estimated by using RAxML with Le-Gascuel (LG) model. Reliability of internal nodes was evaluated by 100 bootstrap replicates. Only bootstrap values ≥50 are shown. Subfamilies that are delineated based on hymenoptera *GR* phylogeny are indicated by brackets. Subfamilies showing interesting evolutionary patterns are named after the orthologs in *N. vitripennis* and *A. mellifera*. The other subfamilies are named as *A*–*H*. All *D. melanogaster* and *T. castaneum* genes are highlighted in blue. Confidently resolved relationships among hymenopteran and non-hymenopteran *Gr* genes are indicated by red. The clade of Grs encoding carbon dioxide receptor is indicated by blue.
(EPS)

Figure S8 Phylogenetic relationships of Hymenoptera *Ir* genes. The same tree as in Figure 4 is shown with gene names. Only bootstrap values ≥50 are shown. Supported subfamilies are indicated by brackets, and named after the orthologs in *L. humile* and *P. barbatus*.
(EPS)

Figure S9 Phylogenetic relationships of *Ir* genes in representative insects. A maximum-likelihood tree of *Ir* genes from *D. melanogaster*, *T. castaneum*, and six hymenopteran species. The topology is estimated by using RAxML with Le-Gascuel (LG) model. Reliability of internal nodes was evaluated by 100 bootstrap replicates. Only bootstrap values ≥50 are shown. Subfamilies that are delineated based on hymenoptera *IR* phylogeny are indicated by brackets, and named after the orthologs in *L. humile* and *P. barbatus*. All *D. melanogaster* and *T. castaneum* genes are highlighted in blue. Confidently resolved relationships among hymenopteran and non-hymenopteran *Ir* genes are indicated by red.
(EPS)

Figure S10 Pairwise comparisons of whole transcriptome between castes for *C. floridanus* and *H. saltator*. Chemosensory receptor genes were highlighted in red.
(EPS)

Table S1 Sequence divergence of chemosensory receptor genes.
(DOCX)

Table S2 Summary of ant antennal transcriptome data sets and mapping results.
(DOCX)

Table S3 Expression patterns of (co-)orthologous *Or* genes in *C. floridanus* and *H. saltator*.
(XLS)

Table S4 List of the 73 odors screened in this study.
(XLS)

Table S5 Alternative strategies for bioinformatic processing of ant transcriptomes do not significantly affect read mapping.
(DOCX)

## References

[1] Moreau CS, Bell CD, Vila R, Archibald SB, Pierce NE (2006) Phylogeny of the ants: diversification in the age of angiosperms. Science 312: 101–104. 16601190 10.1126/science.1124891

[2] Hölldobler B, Wilson EO (1990) The ants. Cambridge, Mass.: Belknap Press of Harvard University Press. xii, 732 p., 724 p. of plates p.

[3] Davidson DW, Cook SC, Snelling RR, Chua TH (2003) Explaining the abundance of ants in lowland tropical rainforest canopies. Science 300: 969–972. 12738862 10.1126/science.1082074

[4] Davidson DW (1997) The role of resource imbalances in the evolutionary ecology of tropical arboreal ants. Biol J Linn Soc 61: 153–181.

[5] Bolton B (2003) Synopsis and Classification of Formicidae. Mem Am Entomol Inst 71: 1–370.

[6] Wilson EO (1976) Which are the most prevalent ant genera? Stud Entomol 19: 187–200.

[7] Moore D, Liebig J (2010) Mechanisms of social regulation change across colony development in an ant. BMC Evol Biol 10: 328. 20977775 10.1186/1471-2148-10-328 PMC2978225

[8] Endler A, Liebig J, Hölldobler B (2006) Queen fertility, egg marking and colony size in the ant *Camponotus floridanus* . Behav Ecol Sociobiol 59: 490–499.

[9] Endler A, Liebig J, Schmitt T, Parker JE, Jones GR, et al (2004) Surface hydrocarbons of queen eggs regulate worker reproduction in a social insect. Proc Natl Acad Sci U S A 101: 2945–2950. 14993614 10.1073/pnas.0308447101 PMC365725

[10] Endler A, Hölldobler B, Liebig J (2007) Lack of physical policing and fertility cues in egg-laying workers of the ant *Camponotus floridanus* . Anim Behav 74: 1171–1180.

[11] Peeters C, Liebig J, Hölldobler B (2000) Sexual reproduction by both queens and workers in the ponerine ant Harpegnathos saltator. Insect Soc 47: 325–332.

[12] Peeters C, Hölldobler B (1995) Reproductive cooperation between queens and their mated workers: the complex life history of an ant with a valuable nest. Proc Natl Acad Sci U S A 92: 10977–10979. 11607589 10.1073/pnas.92.24.10977 PMC40553

[13] Liebig J, Peeters C, Hölldobler B (1999) Worker policing limits the number of reproductives in a ponerine ant. Proc R Soc B 266: 1865–1870.

[14] Liang ZS, Nguyen T, Mattila HR, Rodriguez-Zas SL, Seeley TD, et al (2012) Molecular determinants of scouting behavior in honey bees. Science 335: 1225–1228. 22403390 10.1126/science.1213962

[15] Robertson HM, Warr CG, Carlson JR (2003) Molecular evolution of the insect chemoreceptor gene superfamily in Drosophila melanogaster. Proc Natl Acad Sci U S A 100 Suppl 2: 14537–14542. 14608037 10.1073/pnas.2335847100 PMC304115

[16] Gao Q, Chess A (1999) Identification of candidate Drosophila olfactory receptors from genomic DNA sequence. Genomics 60: 31–39. 10458908 10.1006/geno.1999.5894

[17] Clyne PJ, Warr CG, Freeman MR, Lessing D, Kim J, et al (1999) A novel family of divergent seven-transmembrane proteins: candidate odorant receptors in Drosophila. Neuron 22: 327–338. 10069338 10.1016/s0896-6273(00)81093-4

[18] Vosshall LB, Amrein H, Morozov PS, Rzhetsky A, Axel R (1999) A spatial map of olfactory receptor expression in the Drosophila antenna. Cell 96: 725–736. 10089887 10.1016/s0092-8674(00)80582-6

[19] Fox AN, Pitts RJ, Robertson HM, Carlson JR, Zwiebel LJ (2001) Candidate odorant receptors from the malaria vector mosquito Anopheles gambiae and evidence of down-regulation in response to blood feeding. Proc Natl Acad Sci U S A 98: 14693–14697. 11724964 10.1073/pnas.261432998 PMC64743

[20] Scott K, Brady R Jr, Cravchik A, Morozov P, Rzhetsky A, et al (2001) A chemosensory gene family encoding candidate gustatory and olfactory receptors in Drosophila. Cell 104: 661–673. 11257221 10.1016/s0092-8674(01)00263-x

[21] Dunipace L, Meister S, McNealy C, Amrein H (2001) Spatially restricted expression of candidate taste receptors in the Drosophila gustatory system. Curr Biol 11: 822–835. 11516643 10.1016/s0960-9822(01)00258-5

[22] Clyne PJ, Warr CG, Carlson JR (2000) Candidate taste receptors in Drosophila. Science 287: 1830–1834. 10710312 10.1126/science.287.5459.1830

[23] Wang L, Han X, Mehren J, Hiroi M, Billeter J-C, et al (2011) Hierarchical chemosensory regulation of male-male social interactions in *Drosophila* . Nat Neurosci 14: 757–U392. 21516101 10.1038/nn.2800 PMC3102769

[24] Benton R, Vannice KS, Gomez-Diaz C, Vosshall LB (2009) Variant ionotropic glutamate receptors as chemosensory receptors in Drosophila. Cell 136: 149–162. 19135896 10.1016/j.cell.2008.12.001 PMC2709536

[25] Abuin L, Bargeton B, Ulbrich MH, Isacoff EY, Kellenberger S, et al (2011) Functional architecture of olfactory ionotropic glutamate receptors. Neuron 69: 44–60. 21220098 10.1016/j.neuron.2010.11.042 PMC3050028

[26] Croset V, Rytz R, Cummins SF, Budd A, Brawand D, et al (2010) Ancient protostome origin of chemosensory ionotropic glutamate receptors and the evolution of insect taste and olfaction. PLoS Genet 6: e1001064 doi:10.1371/journal.pgen.1001064. 20808886 10.1371/journal.pgen.1001064 PMC2924276

[27] Hill CA, Fox AN, Pitts RJ, Kent LB, Tan PL, et al (2002) G protein-coupled receptors in Anopheles gambiae. Science 298: 176–178. 12364795 10.1126/science.1076196

[28] Robertson HM, Gadau J, Wanner KW (2010) The insect chemoreceptor superfamily of the parasitoid jewel wasp Nasonia vitripennis. Insect Mol Biol 19 Suppl 1: 121–136. 10.1111/j.1365-2583.2009.00979.x 20167023

[29] Larsson MC, Domingos AI, Jones WD, Chiappe ME, Amrein H, et al (2004) Or83b encodes a broadly expressed odorant receptor essential for Drosophila olfaction. Neuron 43: 703–714. 15339651 10.1016/j.neuron.2004.08.019

[30] Benton R, Sachse S, Michnick SW, Vosshall LB (2006) Atypical membrane topology and heteromeric function of Drosophila odorant receptors in vivo. PLoS Biol 4: e20 doi:10.1371/journal.pbio.0040020. 16402857 10.1371/journal.pbio.0040020 PMC1334387

[31] Krieger J, Klink O, Mohl C, Raming K, Breer H (2003) A candidate olfactory receptor subtype highly conserved across different insect orders. J Comp Physiol A Neuroethol Sens Neural Behav Physiol 189: 519–526. 12827420 10.1007/s00359-003-0427-x

[32] Sato K, Pellegrino M, Nakagawa T, Nakagawa T, Vosshall LB, et al (2008) Insect olfactory receptors are heteromeric ligand-gated ion channels. Nature 452: 1002–1006. 18408712 10.1038/nature06850

[33] Wicher D, Schafer R, Bauernfeind R, Stensmyr MC, Heller R, et al (2008) Drosophila odorant receptors are both ligand-gated and cyclic-nucleotide-activated cation channels. Nature 452: 1007–1011. 18408711 10.1038/nature06861

[34] Smart R, Kiely A, Beale M, Vargas E, Carraher C, et al (2008) Drosophila odorant receptors are novel seven transmembrane domain proteins that can signal independently of heterotrimeric G proteins. Insect Biochem Mol Biol 38: 770–780. 18625400 10.1016/j.ibmb.2008.05.002

[35] Jones PL, Pask GM, Rinker DC, Zwiebel LJ (2011) Functional agonism of insect odorant receptor ion channels. Proc Natl Acad Sci U S A 108: 8821–8825. 21555561 10.1073/pnas.1102425108 PMC3102409

[36] Pask GM, Jones PL, Rutzler M, Rinker DC, Zwiebel LJ (2011) Heteromeric anopheline odorant receptors exhibit distinct channel properties. PLoS ONE 6: e28774 doi:10.1371/journal.pone.0028774. 22174894 10.1371/journal.pone.0028774 PMC3235152

[37] Dahanukar A, Foster K, van der Goes van Naters WM, Carlson JR (2001) A Gr receptor is required for response to the sugar trehalose in taste neurons of Drosophila. Nat Neurosci 4: 1182–1186. 11704765 10.1038/nn765

[38] Ueno K, Ohta M, Morita H, Mikuni Y, Nakajima S, et al (2001) Trehalose sensitivity in Drosophila correlates with mutations in and expression of the gustatory receptor gene Gr5a. Curr Biol 11: 1451–1455. 11566105 10.1016/s0960-9822(01)00450-x

[39] Moon SJ, Kottgen M, Jiao Y, Xu H, Montell C (2006) A taste receptor required for the caffeine response in vivo. Curr Biol 16: 1812–1817. 16979558 10.1016/j.cub.2006.07.024

[40] Bray S, Amrein H (2003) A putative Drosophila pheromone receptor expressed in male-specific taste neurons is required for efficient courtship. Neuron 39: 1019–1029. 12971900 10.1016/s0896-6273(03)00542-7

[41] Wang L, Han X, Mehren J, Hiroi M, Billeter JC, et al (2011) Hierarchical chemosensory regulation of male-male social interactions in Drosophila. Nat Neurosci 14: 757–762. 21516101 10.1038/nn.2800 PMC3102769

[42] Miyamoto T, Amrein H (2008) Suppression of male courtship by a Drosophila pheromone receptor. Nat Neurosci 11: 874–876. 18641642 10.1038/nn.2161 PMC5655991

[43] Jones WD, Cayirlioglu P, Kadow IG, Vosshall LB (2007) Two chemosensory receptors together mediate carbon dioxide detection in Drosophila. Nature 445: 86–90. 17167414 10.1038/nature05466

[44] Lu T, Qiu YT, Wang G, Kwon JY, Rutzler M, et al (2007) Odor coding in the maxillary palp of the malaria vector mosquito Anopheles gambiae. Curr Biol 17: 1533–1544. 17764944 10.1016/j.cub.2007.07.062 PMC3113458

[45] Thorne N, Amrein H (2008) Atypical expression of Drosophila gustatory receptor genes in sensory and central neurons. J Comp Neurol 506: 548–568. 18067151 10.1002/cne.21547

[46] Bonasio R, Zhang G, Ye C, Mutti NS, Fang X, et al (2010) Genomic comparison of the ants Camponotus floridanus and Harpegnathos saltator. Science 329: 1068–1071. 20798317 10.1126/science.1192428 PMC3772619

[47] Smith CD, Zimin A, Holt C, Abouheif E, Benton R, et al (2011) Draft genome of the globally widespread and invasive Argentine ant (Linepithema humile). Proc Natl Acad Sci U S A 108: 5673–5678. 21282631 10.1073/pnas.1008617108 PMC3078359

[48] Smith CR, Smith CD, Robertson HM, Helmkampf M, Zimin A, et al (2011) Draft genome of the red harvester ant Pogonomyrmex barbatus. Proc Natl Acad Sci U S A 108: 5667–5672. 21282651 10.1073/pnas.1007901108 PMC3078412

[49] Pitts RJ, Rinker DC, Jones PL, Rokas A, Zwiebel LJ (2011) Transcriptome profiling of chemosensory appendages in the malaria vector Anopheles gambiae reveals tissue- and sex-specific signatures of odor coding. BMC Genomics 12: 271. 21619637 10.1186/1471-2164-12-271 PMC3126782

[50] Robertson HM, Wanner KW (2006) The chemoreceptor superfamily in the honey bee, Apis mellifera: expansion of the odorant, but not gustatory, receptor family. Genome Res 16: 1395–1403. 17065611 10.1101/gr.5057506 PMC1626641

[51] Engsontia P, Sanderson AP, Cobb M, Walden KK, Robertson HM, et al (2008) The red flour beetle's large nose: an expanded odorant receptor gene family in Tribolium castaneum. Insect Biochem Mol Biol 38: 387–397. 18342245 10.1016/j.ibmb.2007.10.005

[52] Nozawa M, Nei M (2007) Evolutionary dynamics of olfactory receptor genes in Drosophila species. Proc Natl Acad Sci U S A 104: 7122–7127. 17438280 10.1073/pnas.0702133104 PMC1855360

[53] Penalva-Arana DC, Lynch M, Robertson HM (2009) The chemoreceptor genes of the waterflea Daphnia pulex: many Grs but no Ors. BMC Evol Biol 9: 79. 19383158 10.1186/1471-2148-9-79 PMC2680840

[54] Smadja C, Shi P, Butlin RK, Robertson HM (2009) Large gene family expansions and adaptive evolution for odorant and gustatory receptors in the pea aphid, Acyrthosiphon pisum. Mol Biol Evol 26: 2073–2086. 19542205 10.1093/molbev/msp116

[55] Kent LB, Robertson HM (2009) Evolution of the sugar receptors in insects. BMC Evol Biol 9: 41. 19226470 10.1186/1471-2148-9-41 PMC2667405

[56] Slone J, Daniels J, Amrein H (2007) Sugar receptors in Drosophila. Curr Biol 17: 1809–1816. 17919910 10.1016/j.cub.2007.09.027 PMC2078200

[57] Dahanukar A, Lei YT, Kwon JY, Carlson JR (2007) Two Gr genes underlie sugar reception in Drosophila. Neuron 56: 503–516. 17988633 10.1016/j.neuron.2007.10.024 PMC2096712

[58] Jiao Y, Moon SJ, Montell C (2007) A Drosophila gustatory receptor required for the responses to sucrose, glucose, and maltose identified by mRNA tagging. Proc Natl Acad Sci U S A 104: 14110–14115. 17715294 10.1073/pnas.0702421104 PMC1955822

[59] Kwon JY, Dahanukar A, Weiss LA, Carlson JR (2007) The molecular basis of CO2 reception in Drosophila. Proc Natl Acad Sci U S A 104: 3574–3578. 17360684 10.1073/pnas.0700079104 PMC1805529

[60] Robertson HM, Kent LB (2009) Evolution of the gene lineage encoding the carbon dioxide receptor in insects. J Insect Sci 9: 19. 19613462 10.1673/031.009.1901 PMC3011840

[61] Kleineidam C, Tautz J (1996) Perception of carbon dioxide and other “air-condition” parameters in the leaf cutting ant *Atta cephalotes* . Naturwissenschaften 83: 566–568.

[62] De Bie T, Cristianini N, Demuth JP, Hahn MW (2006) CAFE: a computational tool for the study of gene family evolution. Bioinformatics 22: 1269–1271. 16543274 10.1093/bioinformatics/btl097

[63] Chen K, Durand D, Farach-Colton M (2000) NOTUNG: a program for dating gene duplications and optimizing gene family trees. J Comput Biol 7: 429–447. 11108472 10.1089/106652700750050871

[64] Hahn MW, De Bie T, Stajich JE, Nguyen C, Cristianini N (2005) Estimating the tempo and mode of gene family evolution from comparative genomic data. Genome Res 15: 1153–1160. 16077014 10.1101/gr.3567505 PMC1182228

[65] Vosshall LB, Wong AM, Axel R (2000) An olfactory sensory map in the fly brain. Cell 102: 147–159. 10943836 10.1016/s0092-8674(00)00021-0

[66] Wang G, Carey AF, Carlson JR, Zwiebel LJ (2010) Molecular basis of odor coding in the malaria vector mosquito Anopheles gambiae. Proc Natl Acad Sci U S A 107: 4418–4423. 20160092 10.1073/pnas.0913392107 PMC2840125

[67] Burdock GA (1997) Encyclopedia of food and color additives. Boca Raton: CRC Press.

[68] Khan IA, Abourashed EA (2010) Leung's encyclopedia of common natural ingredients: used in food, drugs, and cosmetics. Hoboken, N.J.: John Wiley & Sons, Inc. xxix, 810 p. p.

[69] Sánchez-Gracia A, Vieira FG, Almeida FC, Rozas J (2011) Comparative Genomics of the Major Chemosensory Gene Families in Arthropods. eLS. Chichester: John Wiley & Sons Ltd.

[70] Gadau J, Helmkampf M, Nygaard S, Roux J, Simola DF, et al (2012) The genomic impact of 100 million years of social evolution in seven ant species. Trends Genet 28: 14–21. 21982512 10.1016/j.tig.2011.08.005 PMC3314025

[71] Nei M, Niimura Y, Nozawa M (2008) The evolution of animal chemosensory receptor gene repertoires: roles of chance and necessity. Nat Rev Genet 9: 951–963. 19002141 10.1038/nrg2480

[72] Xu G, Ma H, Nei M, Kong H (2009) Evolution of F-box genes in plants: different modes of sequence divergence and their relationships with functional diversification. Proc Natl Acad Sci U S A 106: 835–840. 19126682 10.1073/pnas.0812043106 PMC2630105

[73] Urbani CB, Boyan GS, Blarer A, Billen J, Ali TMM (1994) A Novel Mechanism for Jumping in the Indian Ant Harpegnathos Saltator (Jerdon) (Formicidae, Ponerinae). Experientia 50: 63–71.

[74] Liebig J, Heinze J, Hölldobler B (1997) Trophallaxis and aggression in the ponerine ant, Ponera coarctata: Implications for the evolution of liquid food exchange in the Hymenoptera. Ethology 103: 707–722.

[75] Sato K, Tanaka K, Touhara K (2011) Sugar-regulated cation channel formed by an insect gustatory receptor. Proc Natl Acad Sci U S A 108: 11680–11685. 21709218 10.1073/pnas.1019622108 PMC3136286

[76] Zube C, Rossler W (2008) Caste- and sex-specific adaptations within the olfactory pathway in the brain of the ant Camponotus floridanus. Arthropod Struct Dev 37: 469–479. 18621145 10.1016/j.asd.2008.05.004

[77] Hoyer SC, Liebig J, Rössler W (2005) Biogenic amines in the ponerine ant Harpegnathos saltator: serotonin and dopamine immunoreactivity in the brain. Arthropod Struct Dev 34: 429–440.

[78] Nakanishi A, Nishino H, Watanabe H, Yokohari F, Nishikawa M (2010) Sex-specific antennal sensory system in the ant Camponotus japonicus: glomerular organizations of antennal lobes. J Comp Neurol 518: 2186–2201. 20437523 10.1002/cne.22326

[79] Ozaki M, Wada-Katsumata A, Fujikawa K, Iwasaki M, Yokohari F, et al (2005) Ant nestmate and non-nestmate discrimination by a chemosensory sensillum. Science 309: 311–314. 15947139 10.1126/science.1105244

[80] Nakanishi A, Nishino H, Watanabe H, Yokohari F, Nishikawa M (2009) Sex-specific antennal sensory system in the ant Camponotus japonicus: structure and distribution of sensilla on the flagellum. Cell Tissue Res 338: 79–97. 19763622 10.1007/s00441-009-0863-1

[81] Dobritsa AA, van der Goes van Naters W, Warr CG, Steinbrecht RA, Carlson JR (2003) Integrating the molecular and cellular basis of odor coding in the Drosophila antenna. Neuron 37: 827–841. 12628173 10.1016/s0896-6273(03)00094-1

[82] Goldman AL, Van der Goes van Naters W, Lessing D, Warr CG, Carlson JR (2005) Coexpression of two functional odor receptors in one neuron. Neuron 45: 661–666. 15748842 10.1016/j.neuron.2005.01.025

[83] Fishilevich E, Vosshall LB (2005) Genetic and functional subdivision of the Drosophila antennal lobe. Curr Biol 15: 1548–1553. 16139209 10.1016/j.cub.2005.07.066

[84] Couto A, Alenius M, Dickson BJ (2005) Molecular, anatomical, and functional organization of the Drosophila olfactory system. Curr Biol 15: 1535–1547. 16139208 10.1016/j.cub.2005.07.034

[85] Wanner KW, Nichols AS, Walden KK, Brockmann A, Luetje CW, et al (2007) A honey bee odorant receptor for the queen substance 9-oxo-2-decenoic acid. Proc Natl Acad Sci U S A 104: 14383–14388. 17761794 10.1073/pnas.0705459104 PMC1964862

[86] Hölldobler B (1966) Futterverteilung durch Männchen im Ameisenstaat. Z Vergl Physiol 52: 430–455.

[87] Erler F, Ulug I, Yalcinkaya B (2006) Repellent activity of five essential oils against Culex pipiens. Fitoterapia 77: 491–494. 16890387 10.1016/j.fitote.2006.05.028

[88] Prajapati V, Tripathi AK, Aggarwal KK, Khanuja SP (2005) Insecticidal, repellent and oviposition-deterrent activity of selected essential oils against Anopheles stephensi, Aedes aegypti and Culex quinquefasciatus. Bioresour Technol 96: 1749–1757. 16051081 10.1016/j.biortech.2005.01.007

[89] Munoz-Torres MC, Reese JT, Childers CP, Bennett AK, Sundaram JP, et al (2011) Hymenoptera Genome Database: integrated community resources for insect species of the order Hymenoptera. Nucleic Acids Res 39: D658–662. 21071397 10.1093/nar/gkq1145 PMC3013718

[90] Altschul SF, Madden TL, Schaffer AA, Zhang J, Zhang Z, et al (1997) Gapped BLAST and PSI-BLAST: a new generation of protein database search programs. Nucleic Acids Res 25: 3389–3402. 9254694 10.1093/nar/25.17.3389 PMC146917

[91] Birney E, Clamp M, Durbin R (2004) GeneWise and Genomewise. Genome Res 14: 988–995. 15123596 10.1101/gr.1865504 PMC479130

[92] Edgar RC (2004) MUSCLE: multiple sequence alignment with high accuracy and high throughput. Nucleic Acids Res 32: 1792–1797. 15034147 10.1093/nar/gkh340 PMC390337

[93] Zdobnov EM, Apweiler R (2001) InterProScan–an integration platform for the signature-recognition methods in InterPro. Bioinformatics 17: 847–848. 11590104 10.1093/bioinformatics/17.9.847

[94] Stamatakis A (2006) RAxML-VI-HPC: maximum likelihood-based phylogenetic analyses with thousands of taxa and mixed models. Bioinformatics 22: 2688–2690. 16928733 10.1093/bioinformatics/btl446

[95] Roshan U, Livesay DR (2006) Probalign: multiple sequence alignment using partition function posterior probabilities. Bioinformatics 22: 2715–2721. 16954142 10.1093/bioinformatics/btl472

[96] Nicholas KB, Nicholas HB, Deerfield DW (1997) GeneDoc: analysis and visualization of genetic variation. EMBNETnews 4: 1–4.

[97] Capella-Gutierrez S, Silla-Martinez JM, Gabaldon T (2009) trimAl: a tool for automated alignment trimming in large-scale phylogenetic analyses. Bioinformatics 25: 1972–1973. 19505945 10.1093/bioinformatics/btp348 PMC2712344

[98] Le SQ, Gascuel O (2008) An improved general amino acid replacement matrix. Mol Biol Evol 25: 1307–1320. 18367465 10.1093/molbev/msn067

[99] Nam J, Nei M (2005) Evolutionary change of the numbers of homeobox genes in bilateral animals. Mol Biol Evol 22: 2386–2394. 16079247 10.1093/molbev/msi229 PMC1464090

[100] Trapnell C, Pachter L, Salzberg SL (2009) TopHat: discovering splice junctions with RNA-Seq. Bioinformatics 25: 1105–1111. 19289445 10.1093/bioinformatics/btp120 PMC2672628

[101] Trapnell C, Williams BA, Pertea G, Mortazavi A, Kwan G, et al (2010) Transcript assembly and quantification by RNA-Seq reveals unannotated transcripts and isoform switching during cell differentiation. Nat Biotechnol 28: 511–515. 20436464 10.1038/nbt.1621 PMC3146043

[102] Brady SG, Schultz TR, Fisher BL, Ward PS (2006) Evaluating alternative hypotheses for the early evolution and diversification of ants. Proc Natl Acad Sci U S A 103: 18172–18177. 17079492 10.1073/pnas.0605858103 PMC1838725

[103] Bohbot JD, Jones PL, Wang G, Pitts RJ, Pask GM, et al (2011) Conservation of indole responsive odorant receptors in mosquitoes reveals an ancient olfactory trait. Chem Senses 36: 149–160. 20956733 10.1093/chemse/bjq105 PMC3020388

