# Supplementary material for: Phylogenetic and Transcriptomic Analysis of Chemosensory Receptors in a Pair of Divergent Ant Species Reveals Sex-Specific Signatures of Odor Coding
Source: PLoS Genet. 2012 Aug 30;8(8):e1002930. doi: 10.1371/journal.pgen.1002930 (PMC3431598; doi:10.1371/journal.pgen.1002930)
Supplement: Table S1 — Sequence divergence of chemosensory receptor genes. (DOCX) [file pgen.1002930.s016.docx]

**Table S1:** Sequence divergence of chemosensory receptor genes.

| Gene family | | Average distance* | Average protein sequence identity |
| --- | --- | --- | --- |
| OR | Overall | 2.558 | 19.45% |
|  | Orco | 0.239 | 84.09% |
|  | A | 0.865 | 47.43% |
|  | B | 0.705 | 62.40% |
|  | C | 0.673 | 64.33% |
|  | D | 1.071 | 46.01% |
|  | E | 1.427 | 36.14% |
|  | F | 1.492 | 33.51% |
|  | G | 1.165 | 39.05% |
|  | H | 1.345 | 35.29% |
|  | 9-exon | 1.902 | 27.44% |
|  | I | 0.749 | 52.01% |
|  | J | 1.053 | 41.17% |
|  | K | 0.479 | 68.77% |
|  | L | 1.308 | 36.11% |
|  | M | 1.027 | 45.59% |
|  | N | 1.092 | 43.12% |
|  | O | 1.555 | 42.40% |
|  | P | 0.961 | 51.29% |
|  | Q | 1.166 | 45.76% |
|  | R | 0.256 | 75.10% |
|  | S | 1.099 | 46.63% |
|  | T | 1.322 | 37.66% |
|  | U | 1.015 | 45.55% |
|  | V | 1.18 | 40.76% |
| GR | Overall | 2.83 | 18.76% |
| IR | Overall | 3.141 | 17.86% |

* average amino acid changes per site, calculated by using MEGA 5.0 and JTT model.
