# Supplementary material for: Phylogenetic and Transcriptomic Analysis of Chemosensory Receptors in a Pair of Divergent Ant Species Reveals Sex-Specific Signatures of Odor Coding
Source: PLoS Genet. 2012 Aug 30;8(8):e1002930. doi: 10.1371/journal.pgen.1002930 (PMC3431598; doi:10.1371/journal.pgen.1002930)
Supplement: Table S2 — Summary of ant antennal transcriptome data sets and mapping results. (DOCX) [file pgen.1002930.s017.docx]

**Table S2:** Summary of ant antennal transcriptome data sets and mapping results.

|  | Castes | Reads type | Total reads (singletons) | Mapped reads |
| --- | --- | --- | --- | --- |
| *C. floridanus* | major worker | 50bp, PE | 94,775,798 | 84,328,152 |
|  | minor worker |  | 67,253,710 | 61,44,66,11 |
|  | male | 50bp, SE  (trim to 34bp) | 33,421,622 | 26,196,896 |
| *H. saltator* | worker | 50bp, PE | 44,588,740 | 40,523,321 |
|  | male | 51bp, SE | 164,320,963 | 106,996,496 |
