# Supplementary material for: Phylogenetic and Transcriptomic Analysis of Chemosensory Receptors in a Pair of Divergent Ant Species Reveals Sex-Specific Signatures of Odor Coding
Source: PLoS Genet. 2012 Aug 30;8(8):e1002930. doi: 10.1371/journal.pgen.1002930 (PMC3431598; doi:10.1371/journal.pgen.1002930)
Supplement: Table S5 — Alternative strategies for bioinformatic processing of ant transcriptomes do not significantly affect read mapping. (DOCX) [file pgen.1002930.s020.docx]

**Table S5:** Alternative strategies for bioinformatic processing of ant transcriptomes do not significantly affect read mapping.

| Species | Caste | Strategy used in manuscript | Alternative strategy | Spearman’s correlation of FPKM values | |
| --- | --- | --- | --- | --- | --- |
|  |  |  |  | Transcriptome wide | Chemosensory genes only |
| *C. floridanus* | Major worker | read1+2  50bp | read1 50bp | 0.995 | 0.996 |
|  |  |  | read1 34bp | 0.990 | 0.991 |
|  |  |  | read2 50bp | 0.995 | 0.997 |
|  |  |  | read2 34bp | 0.990 | 0.991 |
|  | Minor worker | read1+2  50bp | read1 50bp | 0.995 | 0.998 |
|  |  |  | read1 34bp | 0.990 | 0.990 |
|  |  |  | read2 50bp | 0.994 | 0.997 |
|  |  |  | read2 34bp | 0.989 | 0.991 |
|  | Male | 34bp | 50bp | 0.995 | 0.974 |
| *H. saltator* | Worker | read1+2  50bp | read1 50bp | 0.990 | 0.997 |
|  |  |  | read1 34bp | 0.971 | 0.992 |
|  |  |  | read2 50bp | 0.990 | 0.996 |
|  |  |  | read2 34bp | 0.973 | 0.990 |
|  | Male | 50bp | 34bp | 0.980 | 0.982 |
